# A methionine‐lined active site governs carbocation stabilization and product specificity in a bacterial terpene synthase

**DOI:** 10.1002/1873-3468.70325

**Published:** 2026-03-19

**Authors:** Marion Ringel, Carl P. O. Helmer, Shani Zev, Ronja Driller, Emily Buhr, Markus Reinbold, Renana Schwartz, Gabriel Foley, Mikael Boden, Daniel Garbe, Gerhard Schenk, Dan Thomas Major, Bernhard Loll, Thomas Brück

**Affiliations:** ^1^ Werner Siemens‐Chair of Synthetic Biotechnology Technical University of Munich (TUM), TUM School of Natural Sciences Garching Germany; ^2^ Institute of Chemistry and Biochemistry, Laboratory of Structural Biochemistry Freie Universität Berlin Germany; ^3^ Department of Chemistry and Institute for Nanotechnology & Advanced Materials Bar‐Ilan University Ramat‐Gan Israel; ^4^ School of Chemistry and Molecular Biosciences The University of Queensland Brisbane Australia; ^5^ Sustainable Minerals Institute The University of Queensland Brisbane Australia; ^6^ Australian Institute of Bioengineering and Nanotechnology The University of Queensland Brisbane Australia

**Keywords:** biocatalysis, carbocation, green chemistry, MD simulation, terpene, X‐ray crystallography

## Abstract

Terpene synthases (TPSs) generate complex hydrocarbon scaffolds through carbocationic cyclization cascades that demand precise active‐site control to stabilize reactive intermediates. While π–cation and electrostatic interactions are established stabilizing factors, the role of methionine has remained unclear. Here, we identify a methionine‐rich active site in hydropyrene synthase (HpS), a bacterial Class I TPS involved in pseudopterosin biosynthesis. Crystallography, mutagenesis, and multiscale QM/MM simulations reveal that methionine residues provide steric guidance and direct sulfur–carbocation stabilization during catalysis. Mutations alter product distributions, confirming functional relevance. Quantum chemical calculations indicate that sulfur–carbocation interactions are energetically comparable to π–carbocation interactions. These results uncover a previously unrecognized mechanism of carbocation stabilization in terpene biosynthesis.

## Graphical abstract

This study reveals a unique active site enriched in methionine residues and demonstrates that these residues play a critical role by stabilizing carbocation intermediates through novel sulfur–cation interactions. Structure‐guided mutagenesis further revealed variants with significantly altered product profiles, enhancing pseudopterosin formation. These findings pave the way for sustainable, commercially viable production pathways for pseudopterosins and their derivatives.

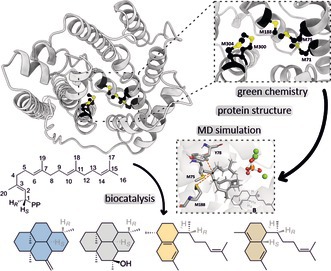

## Abbreviations

**AHD**, alendronate

**GGDP**, *E*,*E*,*E*‐geranylgeranyl diphosphate

**GLDP**, geranyllinalyl diphosphate

**HP**, hydropyrene

**HPol**, hydropyrenol

**HpS**, hydropyrene synthase

**IE A**, isoelisabethatriene A

**IE B**, isoelisabethatriene B

**Ps**, pseudopterosins

**QM/MM**, multiscale quantum mechanics‐molecular mechanics

**SdS**, selina‐4(15),7(11)‐diene synthase

**TPS**, terpene synthase

Terpenoids constitute the most diverse family of natural products, comprising over 80 000 structurally distinct compounds [1, 2, 3, 4] with a broad range of biological activities, including anti‐inflammatory, antimicrobial, and anticancer effects [5]. Their chemical diversity arises from terpene synthases (TPSs), enzymes that catalyze the complex cyclization of linear isoprenoid precursors into polycyclic hydrocarbon frameworks. In Class I diterpene synthases, this transformation begins with the ionization of geranylgeranyl diphosphate (GGDP), releasing a reactive allylic carbocation that initiates a cascade of rearrangements within a hydrophobic, conformationally restricted active site.

Carbocations are intrinsically reactive, and enzymatic stabilization is critical for ensuring productive catalysis. Traditional models of TPS catalysis emphasize the role of π–cation interactions with aromatic residues, charge‐dipole interactions with polar side chains, and electrostatic interactions with the diphosphate moiety and bound Mg^2+^ ions [1, 6, 7, 8, 9, 10, 11, 12, 13]. However, despite recurring observations of methionine residues in active sites in several TPS crystal structures, their catalytic role has remained speculative. Previous computational studies have hinted at the possibility that methionine sulfur atoms might stabilize carbocations through favorable electrostatic interactions [11], but no direct structural or functional evidence has been reported to date.

**Fig. 1:**
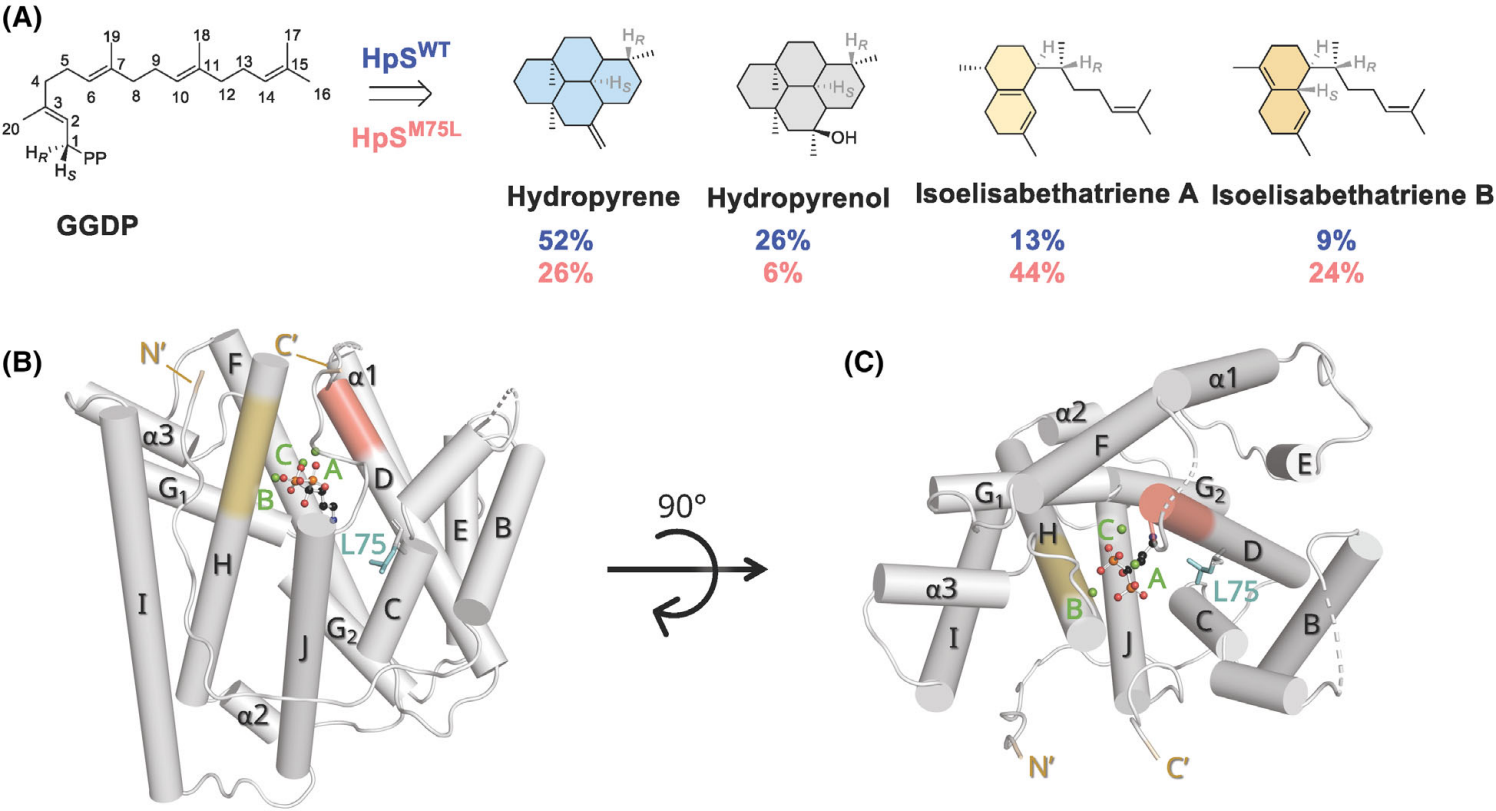
Reaction products of HpS^WT^ and HpS^M75L^ and the structure of HpS^M75L^·Mg^2+^_3_·AHD. (A) In the first step of the reaction, geranylgeranyl diphosphate (GGDP) is converted by HpS to isoelisabethatriene A (IE A), isoelisabethatriene B (IE B), hydropyrene (HP), and hydropyrenol (HPol). Product distribution is given in percentage for HpS^WT^ (blue) and HpS^M75L^ (pink). (B) HpS^M75L^ adopts the classical α‐fold of Class I terpene synthases (TPSs) [70], but it is composed of nine instead of the common 10 core α‐helices. Instead, the 10 residues at the N terminus of HpS^M75L^ adopt a coiled structure that meanders on the protein surface intercalated between α‐helix H and α‐helix I. This position marks the location of the first helix in the α‐helix core of TPSs, which has also been observed in the germacradienol/geosmin synthase [57]. For the structural description, we focus on the protein chain that could be modeled most completely (chain B of HpS^M75L^, Table S2). The closest structural relatives to HpS are shown in (Table S3). Side view of HpS^M75L^ with α‐helices shown as cylinders colored in teal. Arrows indicate the directionality of helices. L75 is shown in stick representation and colored in cyan. The aspartate‐rich motif ^82^DDRAID^87^ is highlighted in red and the ^225^NSE^233^ motif in yellow. Mg^2+^ ions are shown as green spheres. The alendronate (AHD) molecule is presented as a ball and stick model with carbon atoms colored in black, oxygen in red, phosphorous in orange, and nitrogen in blue. (C) Top view of HpS^M75L^ rotated by 90 ° degrees with respect to panel B.

In this study, we present an integrative structural and mechanistic analysis of hydropyrene synthase (HpS), a bacterial Class I TPS from *Streptomyces clavuligerus* involved in the biosynthesis of pseudopterosin precursors. Specifically, pseudopterosins (Ps) belong to the amphilectane‐type diterpene glycoside family with 31 identified members to date, and are promising new drug candidates due to their potent anti‐inflammatory, wound healing and analgesic activities [14, 15]. Production of Ps from their natural source, the Caribbean gorgonian coral *Antillogorgia elisabethae*, is not sustainable or scalable, and total chemical synthesis is not commercially viable [16, 17, 18, 19, 20, 21]. Ps are just one of many examples of highly promising novel drug leads that are hampered from market entry due to the lack of sophisticated, economically feasible and sustainable supply routes [19, 20]. Consequently, alternative production pathways for these bioactive molecules must be developed urgently [21]. Recently, Rinkel *et al*. showed that the Class I TPS hydropyrene synthase from *Streptomyces clavuligerus* (HpS^WT^; WP_003963279.1) produces hydropyrene (HP), hydropyrenol (HPol), isoelisabethatriene A (IE A), and traces of isoelisabethatriene B (IE B) from GGDP (Fig. 1A) [22]. Subsequently, we established the heterologous production of much more significant amounts of the Ps key intermediates IE A and IE B, by engineering HpS from *Streptomyces clavuligerus* (HpS^WT^ to HpS^M75L^) [23]. Synergistic biochemical and homology modeling data of HpS^WT^ suggested the presence of five methionine residues decorating the active site, which are likely to be involved in catalysis [15], yet no crystal structure exists for HpS.

In the current study, we report the first crystal structure of HpS. The crystal structure confirms a novel active‐site architecture, wherein the HpS catalytic pocket is uniquely enriched in methionine residues, which contrasts the aromatic‐rich environments typical of other TPSs [1]. To understand the functional relevance of these residues, we solved the crystal structure of an HpS variant (HpS^M75L^) and performed multiscale QM/MM EnzyDock modeling of the cyclization mechanism in HpS^M75L^ and in a model of the wild‐type enzyme (HpS^WT^). Our modeling results shed light on the stabilization of the intermediate carbocation states and propose that methionine side chains form stabilizing sulfur–carbocation interactions with key intermediates during the reaction cascade, in addition to a traditional steric role. Quantum chemical modeling indicates that sulfur‐based stabilization of carbocations is on par with that provided by π systems. Structure‐guided mutagenesis of these methionine residues dramatically alters the product spectrum, confirming their catalytic significance. These findings reveal a previously unrecognized mode of catalysis in TPSs and suggest that methionine‐rich active sites may constitute a distinct functional motif. Understanding the role of sulfur–carbocation interactions in terpene biosynthesis not only advances our mechanistic understanding of TPS catalysis but also opens new opportunities for the rational engineering of terpene synthases toward valuable natural product scaffolds.

## Methods

### Protein expression and purification

HpS^M75L^ fused to a N‐terminal His_6_‐thioredoxin‐tag (TRX‐tag) in a pETtrx1 vector was expressed in autoinduction media [24] in *Escherichia coli* Rosetta2 DE3 cells. Cells were resuspended in solubilization buffer (50 mm Tris/HCl pH 7.5, 500 mm NaCl, 30 mm imidazole, 5 mm MgCl_2_, and 1 mm DTT) and lysed by sonication. Cell debris was separated from the soluble fraction by centrifugation for 45 min at 55 000 ***g*** in an Avanti J‐26 XP centrifuge (Beckman Coulter, Brea, CA, USA). Target proteins were captured on Ni^2+^‐NTA (Macherey‐Nagel) and washed with solubilization buffer. HpS^M75L^ was eluted with 50 mm Tris/HCl pH 7.5, 500 mm NaCl, 500 mm imidazole, 5 mm MgCl_2_, and 1 mm DTT. The amino‐terminal TRX‐tag of HpS^M75L^ was cleaved by TEV protease while dialyzing in a buffer composed of 20 mm Tris/HCl pH 7.5, 150 mm NaCl, 30 mm imidazole, 5 mm MgCl_2_, and 1 mm DTT overnight. To remove the TRX‐tag and uncleaved protein, the protein was recycled over Ni^2+^‐NTA. The flow‐through was concentrated and applied to size exclusion chromatography (SEC) using a Superdex 75 column (Cytiva, Freiburg, Germany) performed in SEC buffer (20 mm Tris/HCl pH 7.5, 150 mm NaCl, 5 mm MgCl_2_ and 1 mm DTT). Peak fractions were analyzed by SDS/PAGE. Fractions containing the target proteins were pooled. For determination of experimental phases, selenomethionine was incorporated in HpS^M75L^ (SeMet HpS^M75L^). Transformed *E. coli* Rosetta2 DE3 were cultured in minimal media containing selenomethionine [25] and at an OD ~ 0.6, protein expression was induced by addition of 0.5 mm IPTG. Purification was carried out as described above for HpS^M75L^, except that all buffers contained 4 mm DTT.

### Crystallization

Se‐Met HpS^M75L^ was concentrated to 29.4 mg/mL and HpS^M75L^ to 33 mg/mL as measured by the absorbance at 280 nm and co‐crystallized with 1.25‐fold molar excess of alendronate (Alfa Aesar, Germany). Crystals were obtained by the sitting‐drop vapor‐diffusion method at 18 °C. Se‐methionine labelled HpS^M75L^ crystallized with a reservoir solution composed of 0.1 M HEPES/NaOH pH 7.5, 21% (w/v) PEG 10000, and 1% (v/v) 2‐propanol; HpS^M75L^ under a condition composed of 0.1 m Na‐formate and 19% (w/v) PEG 3350. Crystals were cryo‐protected with 25% (v/v) glycerol supplemented to the reservoir solution and subsequently flash‐cooled in liquid nitrogen.

### X‐ray data collection, structure determination, and refinement

Synchrotron diffraction data were collected at the beamline 14.2 of the MX Joint Berlin laboratory at BESSY (Berlin, Germany) and beamline P11 of PETRA III (Deutsches Elektronen Synchrotron, Hamburg, Germany) at 100 K. Diffraction data were processed with XDS [26] (Table S1). Experimental phases were determined by single anomalous dispersion with the program SHELXC/D/E [27] exploiting the SeMet‐HpS^M75L^. The quality of the experimental electron density was further improved with the AUTOSOL routine using PHASER [28] and SOLVE/RESOLVE [29] package in PHENIX [30, 31]. The structure of HpS^M75L^ was determined by molecular replacement. An initial model of Se‐Met HpS^M75L^ was built with the program AUTOBUILD [32] as implemented in PHENIX. The structure was refined by maximum‐likelihood restrained refinement in PHENIX. NCS restrains and secondary structure restrains were applied throughout the refinement. Model building was performed with COOT [33]. Model quality was evaluated with MolProbity [34] and the JCSG validation server (JCSG Quality Control Check v3.1). Secondary structure elements were assigned with DSSP [35]. Figures were prepared using PyMOL (Schrödinger Inc., New York, NY, USA).

### EnzyDock mechanistic docking

Commencing with monomer B, we constructed an all‐atom model of HpS^WT^. The M75L was modeled as M75, and missing residues were added using the Modeller program (residues 38–41 and 90–94) [36]. The diphosphate and metal ions were taken from their holo crystal structure. The binding mode of diphosphate and the metal ions is in standard positions for microbial terpene synthases with all relevant interactions intact [37], and hence, this structure is suitable for modeling. The ligands were docked using EnzyDock, which assures that the structures are in binding modes compatible with the entire catalytic cycle, as detailed below. We performed mechanistic docking using EnzyDock for all intermediate states in routes to both IE and HP (Scheme S1) [38, 39] The EnzyDock program is based on the CHARMM simulation platform [40, 41, 42] and uses simulated annealing molecular dynamics (MD) and Monte Carlo (MC) sampling. We note that similar docking approaches have been proposed [43, 44, 45]. The *mkconf* option was set to true, and the number of conformations was set to 200 for 10 000 MD and 10 000 MC sampling steps. Preliminary docking of all states without any restraints did not result in any consistent pathways using pathfinder that match all biochemical constraints, like regio‐ and stereo‐chemistry, proximity of C1 to O2α, proximity of (de)protonation sites to acid/base pairs, and proximity of stabilizing moieties to carbocations [15]. Subsequently, we employed consensus restraints, which ensure that docked poses adopt poses similar to the docking seed, which for the current project were chosen to be cations I and E' for formation of HP/HPol and IE A/B, respectively. The consensus restraints were applied in sequential mode, such that each state is restrained relative to its immediate mechanistic predecessor. We applied point nuclear Overhauser effect (PNOE) to match the atom's position using consensus force constants of 50 kcal·mol^−1^·Å^−1^, and the PNOEs were applied to selected carbon atoms along the hydrocarbon backbone. Additionally, a set of NOE restraints was employed during docking to ensure correct stereo‐ and regio‐chemistry for all reaction steps (Table S7). To identify matching poses along a reaction path, we used the pathfinder approach [15]. The RMSD criteria between the intermediate states along the reaction coordinate were defined as a linear interpolation between a low RMSD value and a high RMSD value. This interpolation scheme relies on the binding promiscuity obtained from the cluster analysis, which showed that the number of clusters per state increases as a function of reaction progress. The RMSD was 2.3 Å (low) to 2.5 Å (high). Final analysis for each reaction pathway (HP and IE) and (HpS^WT^ and HpS^M75L^) was performed on the model best matching the restraints. Subsequently, we performed QM(M06‐2X) [46]/MM [47] minimization on each intermediate as implemented in EnzyDock [15] The QM/MM energies obtained for the reaction show a gradual decrease in energy, which is typical for TPS reactions and further lends support to the EnzyDock docked structures (Fig. S13). Active‐site volumes were computed as described previously [37].

Model quantum chemical calculations of snapshots from QM/MM simulations (Table S11) employed the M06‐2X/6–31 + G(d,p) level of theory [46] and the Gaussian 16 software [48]. Model quantum chemical calculations on small carbocations (Table S12) employed the ωB97M‐V/def2‐TZVPD level of theory [49] and the Q‐Chem 6.2 software [50]. SAPT2 calculations [51] (Table S13) were also performed using the Q‐Chem 6.2 software.

### Gene cloning, plasmid construction, and culture conditions

DNA manipulations and cloning procedures were performed according to standard protocols. All variants were introduced by QuikChange site‐directed mutagenesis (Table S8). Correctness of the amplified gene sequences was assessed by DNA sequencing. Plasmid generation and cloning was carried out using *E. coli* DH5α strain following standard cloning procedures. The plasmid for production of diterpene precursors as well as diterpenoid products were constructed as previously described [23, 52]. Terpene production was conducted in *E. coli* ER2566 strain. Experiments for terpene production was carried out in modified R‐media [53] [13.3 g·L^−1^ KH_2_PO_4_, 4.0 g·L^−1^ (NH_4_)_2_HPO_4_, 1.7 g·L^−1^ citric acid, 4.88 mL·L^−1^ 1 M MgSO_4_, 2.45 mL·L^−1^ 0.1 m ammonium Fe^3+^ citrate, 1.00 mL·L^−1^ 100× Trace Element solution (5.0 g·L^−1^ EDTA, 84 mg·L^−1^ ZnCl_2_, 13 mg·L^−1^ CuCl_2_*2H_2_O, 10 mg·L^−1^ CoCl_2_*2H_2_O, 10 mg·L^−1^ H_3_BO_3_, 1.6 mg·L^−1^ MnCl_2_*4H_2_O)] supplemented with 30 g·L^−1^ glycerol and 5 g·L^−1^ yeast extract at 22 °C and 120 rpm shaking. Chloramphenicol (35 μg·mL^−1^) and Kanamycin (50 μg·mL^−1^) were added as required.

### Terpene extraction

Cultivation broth was extracted by adding the same volume extraction solution (ethanol, ethyl acetate, and hexane; 1:1:1). The mixture was shaken for 4 h at room temperature and subsequently centrifuged down for 5 min at 8000 ***g*** to separate the organic phase. A sample of the organic phase was analyzed via GC‐FID or GC–MS as appropriate.

### Analytics

Analysis of terpenes was performed using a Trace GC–MS Ultra system with DSQII (Thermo Scientific, Waltham, MA, USA). The sample (1 μL, 1/10 split) was injected by a TriPlus auto sampler onto a SGE BPX5 column (30 m, I.D. 0.25 mm, film 0.25 μm) with an injector temperature of 280 °C. Helium was used as carrier gas with a flow rate of 0.8 mL·min^−1^. Initial oven temperature was set to 50 °C and held for 2 min. The temperature was increased to 320 °C at a rate of 10 °C·min^−1^ and held for 3 min. MS data were recorded at 70 eV (EI) in positive mode in a range between 50 and 650. GC‐FID analysis was carried out accordingly. Quantification of diterpenes was carried out by correlation of the FID peak area to a defined taxadiene standard of known quantity as previously described [52].

### Amino acid sequence analysis

A total of 9336 sequences were downloaded from InterPro if they contained a single TPS family 2, C‐terminal metal binding domain (Pfam ID: PF19086) and if they were labelled with an appropriate TPS‐tag (Table S6). Sequences were clustered at 65% identity using Cd‐hit [54] and aligned with MAFFT FFT‐NS‐1 [55]. This alignment was used to identify candidate sequences that contained methionine residues within the alignment that were 5 amino acids upstream or 5 amino acids downstream of M75 in HpS^WT^ (1210 sequences).

The candidate sequence set was further refined by removing sequences smaller than 300 amino acids, larger than 550 amino acids, or which contained ambiguous amino acid characters—B, J, O, U, X, or Z and then structurally aligned using MAFFT‐DASH [56] (977 sequences). We then identified a subset of sequences that had a methionine aligned with M75 in HpS^WT^ as well as a conserved NSE motif and conserved RY pair (relative to the motif positions N225, S229, E233 and R313, Y314 in HpS^WT^) (333 sequences).

## Results and discussion

In this section, we first mention the product profile shift caused by the M75L mutation, which redirects HpS catalysis from hydropyrene/hydropyrenol toward isoelisabethatriene formation. We then report the crystal structure of HpS^M75L^ with Mg^2+^ and alendronate, revealing an unusually methionine‐enriched active site compared with canonical TPSs. Guided by this structure, we use multiscale QM/MM EnzyDock mechanistic docking to define substrate binding and folding, map key carbocation intermediates, and identify recurring sulfur–carbocation contacts consistent with stabilizing interactions. Subsequently, we quantify sulfur–carbocation interactions using quantum chemistry model calculations. Finally, structure‐guided mutagenesis tests these predictions by showing that targeted substitutions at methionine positions perturb catalytic activity and product branching.

### Product profile changes by M75L mutation

Previous studies have shown that a single M75L mutation shifts the product distribution in HpS from HP and HPol to IE A and IE B [23]. Hence, we begin our studies by solving the crystal structure of the HpS^M75L^ variant.

### Structural analysis of HpS^M75L^ variant

**Fig. 2:**
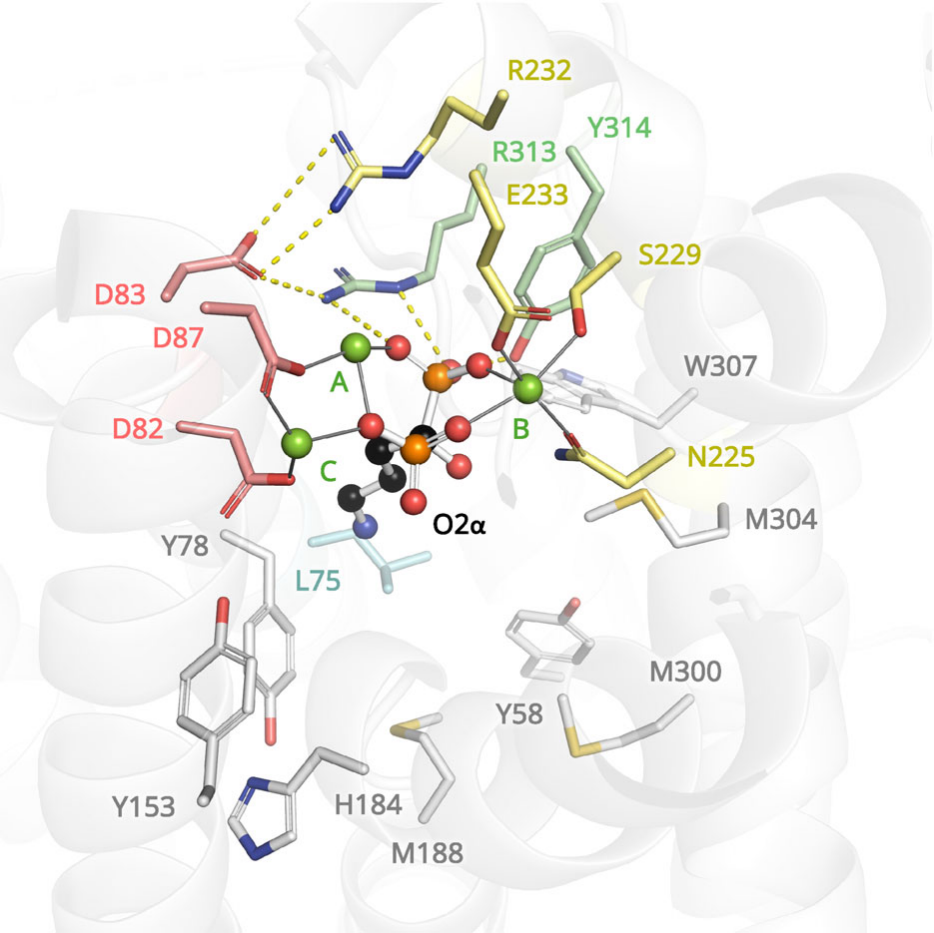
View into the active site of HpS^M75L^. Residues of both metal binding motifs involved in Mg^2+^ coordination are shown in stick representation. Residues of the aspartate‐rich motif are depicted in light red and residues of the NSE motif are colored in yellow with R232 forming a salt bridge to D83. Mg^2+^_C_ is coordinated monodentately by D82 of the aspartate‐rich motif, D87 and one oxygen of the phosphonate group of alendronate (AHD), while Mg^2+^_A_ is coordinated monodentately by the carboxylate groups of D83 and D87 as well as two oxygens located on the diphosphonate moiety of AHD. Mg^2+^_B_ is also coordinated by two oxygen atoms located on the diphosphonate group of AHD, as well as residues N225, S229, and E233 from the NSE motif. The RY pair is colored in gray with R313 forming a salt bridge to D83 and the phosphonate function as well as Y324 to the phosphonate function. Upon binding of AHD, H184 rotates out of the active site, thereby providing space for the propyl‐amino group of AHD. Hydrogen bonds and salt bridges are indicated by yellow dashed lines. Residues involved in Mg^2+^ coordination are linked to the respective Mg^2+^ by a gray line. Aromatic residues and methionine residues lining the active‐site pocket are shown in gray. L75 is drawn in cyan, which is a methionine residue in HpS^WT^. The oxygen labelled O2α is presumable the reactive oxygen attached to the geranylgeranyl moiety of the native substrate geranylgeranyl diphosphate (GGDP).

We determined the crystal structure of HpS^M75L^ in presence of the bisphosphonate inhibitor alendronate (AHD) to 3.04 Å resolution (HpS^M75L^·Mg^2+^_3_·AHD; Figs 1B,C, S1, Table S1, Supplementary text, and Movie S1). AHD mimics the TPS substrate GGDP and can therefore aid in obtaining the closed conformation of HpS. HpS^M75L^ is arranged as a dimer, confirmed by size exclusion chromatography (Fig. S2), with the monomers oriented in an antiparallel manner. HpS features two metal ion binding motifs, facilitating the coordination of three Mg^2+^ ions that are essential for substrate binding (Figs 1B,C, 2, Fig. S3). The aspartate‐rich motif residing in the C‐terminal end of α‐helix D is longer (^82^DDRAID^87^) than the canonical DDXXD motif (Figs 1B,C, 2). Compared with TPSs with a canonical motif (e.g., CotB2), α‐helix D is more extended and hence the secondary structure is arranged more compactly, which allows the C‐terminal residue D87 to be involved in the coordination of Mg^2+^_A_. In contrast, in most TPSs, the canonical motif of the corresponding aspartate is located in a loop region [13]. The second metal ion binding motif, labelled the NSE motif, is comprised of residues ^225^NDLHSFARE^233^ (Figs 1B,C, 2, Fig. S3).

Another conserved motif is the ^313^RY^314^ pair observed in microbial TPSs (Fig. 2, Fig. S3) [37]. Upon the transition from the open to the closed conformation, a bidentate salt bridge is formed between D83 of the aspartate‐rich motif, R232 of the NSE motif and R313 of the RY pair. Consequently, the RY pair moves closer to the catalytic site. Y314 establishes a hydrogen bond to the diphosphonate moiety of AHD. A similar crucial involvement of the RY pair in substrate stabilization has been described for other TPSs [8, 13, 57]. We have previously proposed that W288, located six amino acids upstream of the RY pair in CotB2 [13], is functionally important. In HpS W307 is conserved, having an identical spacing to the RY pair as described previously for CotB2 [13] (Fig. 2, Fig. S3 and Movie S1).

The G_1/2_ helix‐break motif has been demonstrated to facilitate the substrate‐induced fit mechanism of Class I TPSs [58]. The motif is located in a short linker region between α‐helices G_1_ and G_2_ (Fig. 1B,C, Fig. S3) and is characterized by the sequence ^179^RRHGG^183^. Here, R179 acts as the pyrophosphate sensor, which is typical of microbial TPSs [37], and G182 as the linker, while G183 is proposed to serve as the effector in the motif, which causes the release of the diphosphate moiety due to interactions with the commencing allyl carbocation at C1‐C3 of the substrate. Specifically, at the onset of the TPS reaction, R179 is proposed to shift toward the bound GGDP, which leads to a structural reorientation of the G_1/2_ helix‐break motif. This moves the catalytic carbonyl oxygen atom of the effector residue G183 toward the active site and triggers the cleavage of GGDP.

The active site of HpS^M75L^ is decorated by the aromatic residues Y58, Y78, H184, Y153, and W307 (Fig. 2, Fig. S3), although most of these residues' π systems are not oriented to allow π–carbocation stabilization. Moreover, a surprisingly large number of methionine residues point into the active site: L75 (M75 in HpS^WT^), M188, M300, and M304 (Fig. 2). Several of the methionine residues form interactions with π‐rings, and these contacts are known to stabilize protein structures [59, 60]. M300 interacts with Y58, Y189, F296, while M188 interacts with Y78, suggesting that these residues possibly serve structural roles. Previously, we reported that single mutations of several of the active‐site methionine residues have an effect on product formation [15]. While for M188 all introduced mutations led to inactivation, variants of M75, M300, and M304 led to altered product portfolios [15]. The variant HpS^M75L^ caught our attention since it significantly shifted the product portfolio toward the desired IE A (Fig. 1A), the direct precursor of erogorgiaene, and thus the first committed step toward Ps [15]. Hence, the current crystal structure in conjunction with our preliminary mutational studies [15] indicates that these methionine residues may in fact play a direct role in catalysis. This will be expanded further below through modeling and mutagenesis studies.

### Mechanistic docking and the catalytic role of methionine

In the following section, QM/MM EnzyDock docking results are presented, which highlight the structural and energetic basis for the catalytic role of M188 and M304 in stabilizing carbocation intermediates via sulfur–cation interactions. The basic chemical mechanism leading to the formation of HP, HPol, IE A, and IE B is discussed, as well as substrate binding in HpS^WT^ and HpS^M75L^. Furthermore, pathways to hydropyrene, hydropyrenol, and isoelisabethatrienes A and B in HpS are evaluated, followed by an analysis of the energetic basis for sulfur–cation interactions.

Chemical Mechanism of HpS. The HpS‐catalyzed reaction leads to the formation of the products HP, HPol, IE A, and IE B (Fig. 1A) [22], while the M75L mutation drastically alters product distribution, shifting production from HP/HPol to isoelisabethatrienes (IE A and B). To further advance our understanding of this enzyme's mechanism, it is important to investigate the inherent reaction chemistry *in vacuo*. In previous work, we thus studied the competing pathways leading to these products using quantum chemical calculations in the gas phase [61]. We demonstrated that there is a great thermodynamic preference for HP and HPol formation (reaction free energy is ~ − 63 kcal·mol^−1^ using the M062X/6–31 + G(d,p) level of theory), and hence, the biosynthesis of the IE products (reaction free energy is ~ − 35 kcal·mol^−1^ at the same level of theory) is most likely under kinetic control [62].

**Fig. 3:**
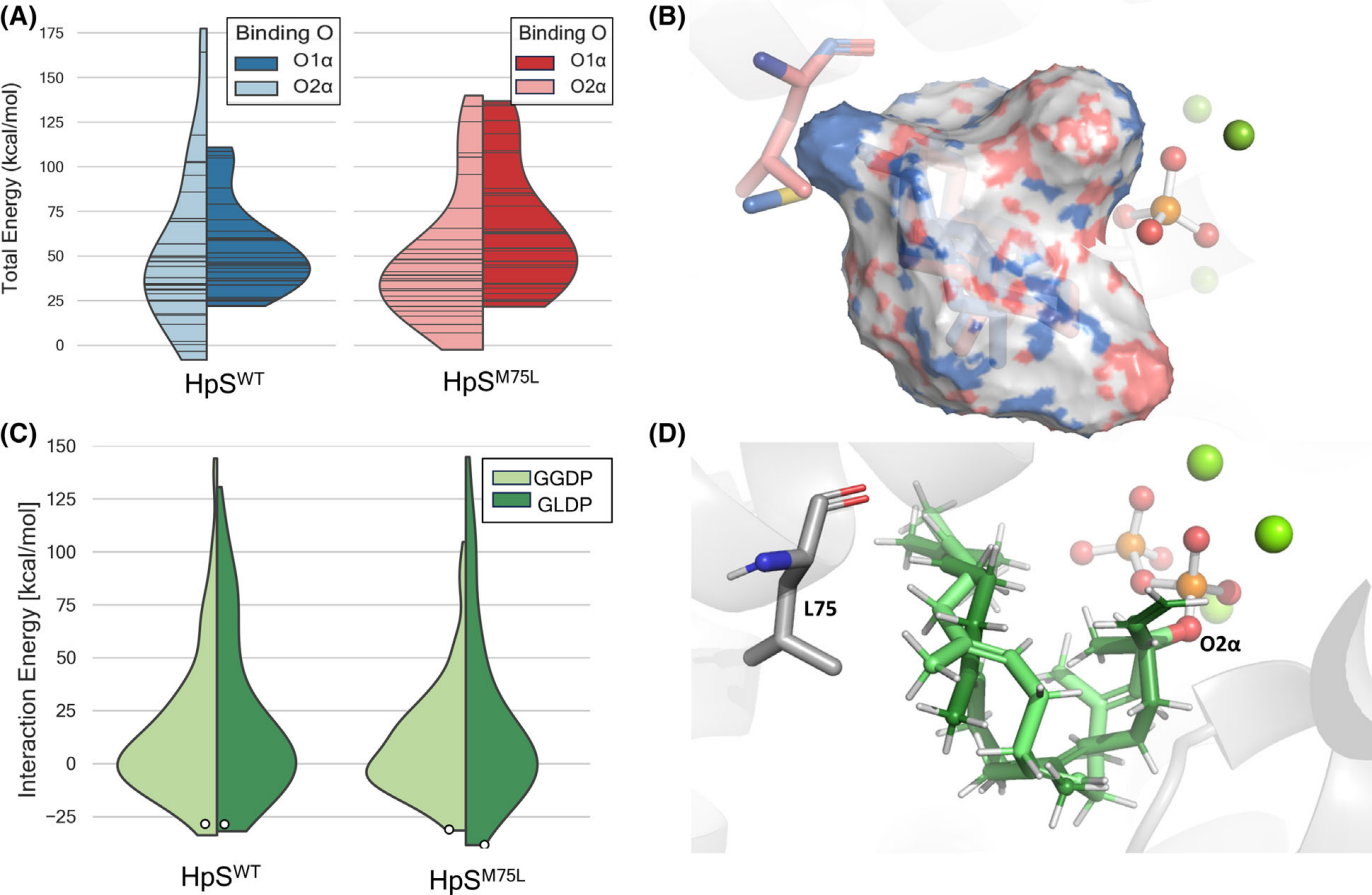
Energy distributions of the docked poses of geranylgeranyl diphosphate (GGDP) and geranyllinalyl diphosphate (GLDP). (A) Oxygen‐isoprenoid binding preferences in HpS^WT^ and HpS^M75L^. In both systems, the preference is for O2α. (B) Active‐site volume in HpS^WT^ and HpS^M75L^. Regions of excess volume are shown as blue (HpS^WT^) and red (HpS^M75L^). (C) Interaction energy distributions of the docked poses of GGDP and GLDP in HpS^WT^ and HpS^M75L^. Docking was performed without any restraints. Selected substrate poses matching those from consensus restrained docking are shown as white circles. (D) Docked product‐like poses of GGDP (light green) and GLDP (dark green) in HpS^WT^ and HpS^M75L^, respectively.

To further investigate the role of HpS in the biosynthesis of HP, HPol, IE A, and IE B, we performed EnzyDock [38] multiscale QM/MM mechanistic docking studies. We docked GGDP and downstream carbocations leading to the final products HP, HPol, IE A, and IE B (Scheme S1) into the active sites of HpS^WT^ and HpS^M75L^. EnzyDock identified preferred binding poses of the substrate and key carbocation intermediates within the active site of HpS [37]. From mechanistic docking, we inferred the most plausible binding mode of the GGDP substrate and intermediates in the active site (Fig. 3). For further details regarding EnzyDock docking, see the Supporting Information.

### Substrate folding and discrimination in favor of hydropyrene or isoelisabethatriene

The HpS^M75L^ crystal structure was resolved with bound AHD, making it challenging to determine which pyrophosphate (PP_i_) oxygen connects the isoprenoid moiety. Hence, we docked the GGDP connected to both O1α and O2α and found a clear energetic preference for binding to O2α for both the HpS^M75L^ crystal structure and the modeled HpS^WT^ structure (Fig. 3A). This agrees with our recent general finding that microbial TPSs bind via O2α whereas plant TPSs bind via O1α [37]. Next, we calculated the active‐site volume of HpS^WT^ and HpS^M75L^, obtaining values of 417 Å^3^ and 407 Å^3^, respectively. The contribution to the excess volume in HpS^WT^ is likely associated with the methyl group at the Cγ position in HpS^M75L^, which protrudes into the active site (see blue surface near the variant site in Fig. 3B). This effect is discussed further below.

In our EnzyDock QM/MM mechanistic docking of routes leading toward IE and HP, we docked the final carbocation intermediate as the docking ‘seed’ and all prior mechanistic states were docked sequentially in a ‘retrosynthesis’ fashion using consensus and additional restraints [38, 63]. This docking process, which is facilitated by EnzyDock, allows identification of mechanistically meaningful poses with correct stereo‐ and regio‐chemistry, even when employing medium‐resolution crystal structures. Using this strategy, the isoprenoid fold of docked poses of GGDP and geranyllinalyl diphosphate (GLDP) are consistent with the formation of the final product. To demonstrate that the mechanistically docked GGDP and GLDP poses also correspond to low energy conformations, free, unrestrained, docking of these species was performed (see white circles in Fig. 3C,D).

An important question is why HpS^WT^ forms mostly HP and HPol while HpS^M75L^ forms mostly IE A and B. The substrate GGDP (where the alkene chain is the (2*E*, 6*E*, 10*E*)‐GG isomer) can undergo isomerization to (S)‐GLDP (where the alkene chain is the (6*E*, 10*E*)‐GG isomer) by virtue of reattachment to the diphosphate moiety via the C3 position. This is likely an equilibrium, wherein the C‐O bond cleavage is slow, while the subsequent ring formation is fast [10, 13, 61].

Hence, following initial C1‐O bond cleavage, the reaction may proceed via one of two scenarios, either via C1‐C10 bond formation toward HP and HPol or reattachment to the diphosphate oxygen atom via C3 toward IE A and B. This shift in product formation due to a single M75L mutation could be due to a preference of HpS^M75L^ for GLDP and HpS^WT^ for GGDP. To quantify whether such a preference exists, we analyzed the docked poses for both GGDP and GLDP in HpS^WT^ and HpS^M75L^. The docked poses energy distributions in HpS^WT^ show a slight preference for GGDP over GLDP (3 kcal·mol^−1^). In contrast, the HpS^M75L^ variant shows a more pronounced preference for GLDP over GGDP (10 kcal·mol^−1^) (Fig. 3C). Inspection of the interactions suggests that this differential preference is due to steric congestion from the side chain in the M75L variant, wherein the methyl group at the Cγ position protrudes into the active site (Fig. 3B,C). This changes the fold of the substrate and shifts the equilibrium toward GLDP in HpS^M75L^. Hence, M75 likely plays a steric role in determining optimal substrate folding in the active site.

### Pathways to hydropyrene and hydropyrenol in HpS

The optimal binding modes of all docked states (*i.e*., GGDP and carbocation intermediates) in HpS^WT^ and HpS^M75L^ are similar and will hence be discussed together. GGDP is partially folded placing C1 in proximity to C10, and the distance between C1 and the *Si*‐face of C10 is 4.6/4.2 Å for HpS^WT^/HpS^M75L^, respectively (Fig. S10). Considering the expected accuracy of our docking, we consider these values as similar. We note that the backbone carbonyl oxygen of G182 interacts with Mg^2+^_C_ via a water molecule and is the linker part of an activator motif identified previously in selina‐4(15),7(11)‐diene synthase (SdS) [58]. In SdS, the analogous residue is D181. Following heterolytic C‐O bond cleavage in GGDP, carbocation B is formed, which may be stabilized by M188 ($R_{C^{+}-S}$ = 4.4/4.1 Å in HpS^WT^/HpS^M75L^), Y78 ($R_{C^{+}-\pi }$ = 5.2/5.4 Å) and M75 ($R_{C^{+}-S}$ = 3.5 Å). The catalytic importance of M188 and Y78 was demonstrated in previous mutagenesis studies [15], and M188 possibly plays a role similar to that of W186 in CotB2, which stabilizes the initial cation and caps off the active site. A hydride transfer converts carbocation B (Scheme S1) to allyl cation C, which is stabilized by Y58 ($R_{C^{+}-O}$ = 4.3/4.5 Å in HpS^WT^/HpS^M75L^) and by M304 ($R_{C^{+}-S}$ = 4.7/4.8 Å in HpS^WT^/HpS^M75L^) and by the diphosphate moiety and D225 via electrostatic control [11] ($R_{C-O}$ = 4.2/4.3 Å in HpS^WT^/HpS^M75L^). The double bond on C14‐C15 then reacts with the cation at C1 to form intermediate D, stabilized by PP_i_, which is also poised to deprotonate the isopropyl hydrocarbon tail by O1α ($R_{C-O1\alpha }$ = 3.0/3.0 Å) or a water molecule. Although no water molecules were modeled in the crystal structure of HpS^M75L^, water molecules coordinating Mg^2+^ ions were added in all docking runs.

Additionally, we performed two independent runs with and without an active‐site water molecule (W_cat_) in our EnzyDock studies of HpS, which is located in a position similar to a water molecule in SdS that interacts with Mg_A_ and D82 [58]. Re‐protonation may occur via PP_i_ at C6 ($R_{C6-O2\alpha }$ = 4.5/4.5 Å), or by water via G183 ($R_{C-O}$ = 3.0/3.0 Å), forming cation F [64]. We stress that this de/re‐protonation likely entails a shuffling of protons as shown by labelling experiments using incubation in D_2_O, such that a solvent proton is stereospecifically incorporated at C6 [22].

**Fig. 4:**
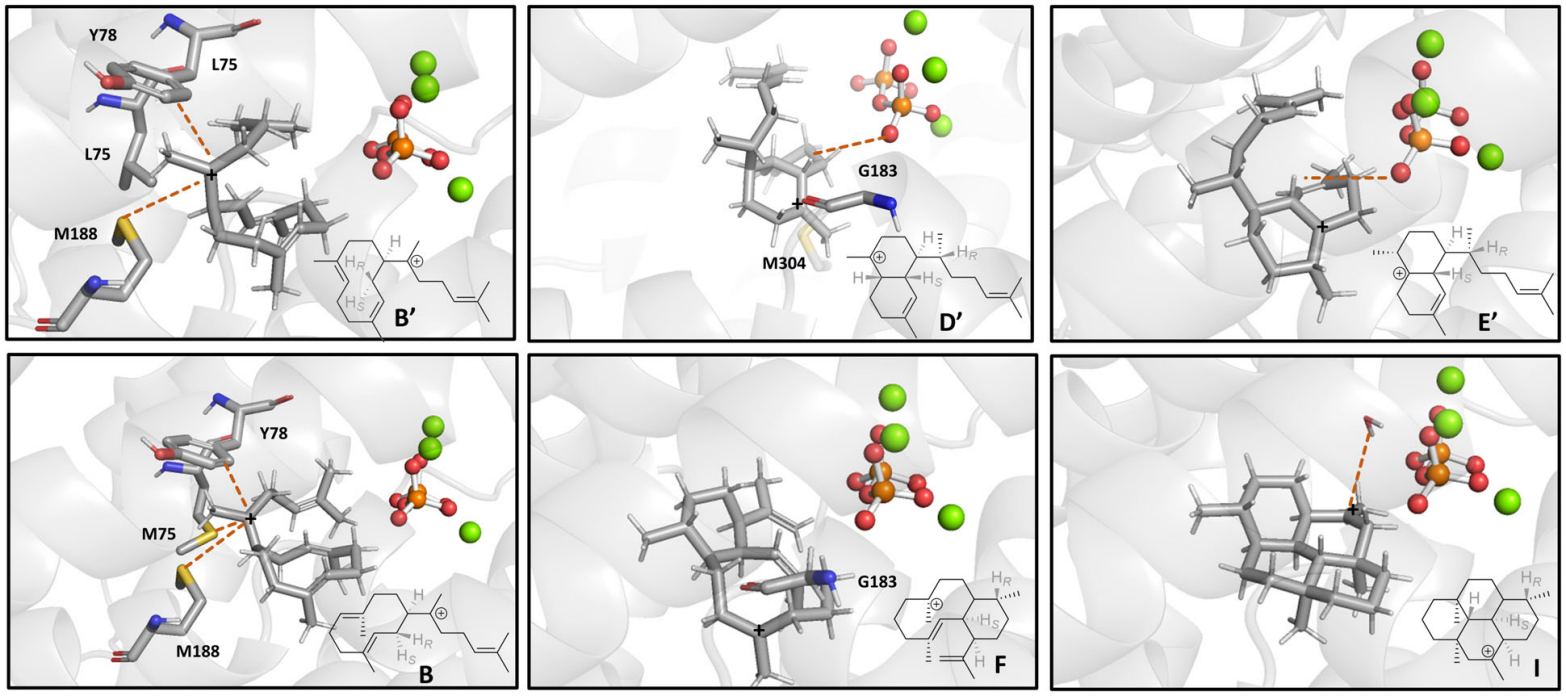
Selected reaction states along the pathway to IE/HP docked into HpS^WT^ and HpS^M75L^ using EnzyDock. Top: pathway to isoelisabethatriene: B′, D′ and E′ docked into HpS^M75L^. Bottom: pathway to hydropyrene: B, F, and I docked into HpS^WT^. Note W_cat_ in proximity to cation I.

The subsequent reaction cascade F → G → I is likely a barrierless process, involving short‐lived intermediate carbocations, as suggested by model quantum chemistry calculations [61]. This is supported by the current QM/MM calculations in HpS using EnzyDock, which predict a spontaneous formation of I from D (Fig. S13a). Deprotonation of I ($R_{C-O1\alpha }$ = 3.0/2.8 Å in HpS^WT^/HpS^M75L^) produces HP, while HPol is formed due to a stereospecific water molecule attack. Such a water molecule (W_cat_) in HpS could participate in formation of Hpol or deprotonation to form HP, either directly or via PP_i_ ($R_{C-W_{\mathrm{cat}}}$ = 4.4/4.5 Å) (Fig. 4).

### Pathways to isoelisabethatrienes a and B in HpS

The GGDP substrate can undergo isomerization to (S)‐GLDP by virtue of reattachment to the diphosphate via the C3 position (Fig. S11), as suggested by Rinkel *et al*. [22]. Rotation around the C2‐C3 bond places the C1 position near the *Si*‐face of C10. Following detachment of the hydrocarbon from PP_i_, carbocation B′ is formed, which may be stabilized by M188 ($R_{C^{+}-S}$ = 4.4/4.1 Å in HpS^WT^/HpS^M75L^) and Y78 ($R_{C^{+}-O}$ = 4.2/3.9 Å in HpS^WT^/HpS^M75L^). As discussed above, the established catalytic importance of M188 and Y78 lends support to the proposed role of these residues in stabilizing reaction intermediates. Allyl cation C′ may be stabilized by the diphosphate moiety emphasizing electrostatic control ($R_{C-O}$ = 5.0/5.0 Å in HpS^WT^/HpS^M75L^) [11]. Cation D′ is stabilized by M304 ($R_{C^{+}-S}$ = 5.1/4.5 Å), and is also poised to be deprotonated at the C6 position ($R_{C6-O1}$ = 3.4/3.8 Å) to form IE B. The deprotonating agent may be O2α, either directly or via the carbonyl group of G183 in conjunction with a water molecule ($R_{\mathrm{CH}-O}$ = 3.5/4.4 Å), which has been proposed to play a similar role in SdS [64]. Cation D′ may transform to E′ via a 1,2‐hydride transfer, and E′ may be deprotonated by PP_i_ group via O2α ($R_{\mathrm{CH}-O}$ = 4.6 Å) to form IE A. According to the current EnzyDock docking studies, the final carbocation intermediates in the HP/HPol and IE A/B pathways superpose nearly perfectly, suggesting that the overall active site supports similar reaction pathways (Fig. S12).

To quantify the pathways toward the final carbocations leading to HP and IE in HpS^WT^ and HpS^M75L^, we performed QM/MM geometry optimizations for the carbocation intermediates obtained from EnzyDock. The energies for these intermediates are shown in Fig. S13 and Tables S9, S10, together with values obtained for the gas‐phase reactions. Similarly, to the gas‐phase reactions, there is a progressive reduction in energy due to the exchange of π‐bonds for σ‐bonds. There are, however, several differences worthy of note. Firstly, the initial carbocation (B and B′) is destabilized compared with the gas‐phase. This so‐called ‘slow‐starter’ effect is due to loss of electrostatic interaction between the formed allyl cation (B and B′) and the PP_i_ moiety [65]. Secondly, the stability of the final carbocations is increased in the enzyme, which is due to the final carbocation being in proximity to the PP_i_ moiety [66, 67]. Thirdly, we note that geometry optimization of F leads directly to I., that is, QM/MM calculations in the WT enzyme predict this reaction to be spontaneous in HpS, like the reaction in the gas phase [61].

### Sulfur–cation interaction

The current crystal structure reveals a methionine‐rich active site, while EnzyDock mechanistic simulations suggest that M188 and M304 play key roles in stabilizing carbocation intermediates. The magnitude of these stabilizing interactions is analyzed in this section.

EnzyDock QM/MM mechanistic docking suggests that the active site in HpS plays a prominent role in stabilizing the cations along the reaction pathway. We observed that the amino acids Y58, M75, M188, Y78, G183, and M304 are likely to play roles in the HpS mechanism, in agreement with mutagenesis studies [15]. Of particular interest is the cation–sulfur interaction through the numerous active‐site methionine residues, and the Met188 sulfur–cation interaction could play an important role in both mechanistic routes. This is a rather unusual interaction in TPSs, which we have previously proposed could play a role in trichodiene synthase [11]. It should be noted that the formation of dative bonds between methionine and carbocations is intrinsically precluded during QM/MM EnzyDock simulations, as the active‐site residues are modeled using a classical mechanical (MM) force field. To evaluate the potential for dative bonding, we conducted QM/MM geometry optimizations for carbocation B′ in which the M188 side chain was explicitly included in the QM region. These calculations did not result in dative bond formation between M188 and carbocation B′. We attribute this observation to the significant steric congestion within the active site, which prevents the proximal orientation and subsequent orbital overlap necessary for dative coordination.

To quantify the strength of methionine‐carbocation interactions at a quantum chemical level, we computed the complexation free energy of both cations (B and B′) with a model methionine residue in the gas phase and continuum chloroform and water based on the docked poses. During minimization in the absence of the full enzyme environment, these complexes formed a dative bond, with distances of ca. 1.9 Å. The complexation energies range from −25.3 kcal·mol^−1^ to −29.1 kcal·mol^−1^ in the gas‐phase and −26.2 kcal·mol^−1^ to −30.1 kcal·mol^−1^ in water (Table S11), underscoring the potency of this type of interaction.

Additionally, we performed model quantum chemistry calculations to compare simple carbocations interactions with sulfur and π systems. We compared the parallel interaction of CH_3_^+^, CH_3_CH_2_^+^, (CH_3_)_2_CH^+^, and (CH_3_)_3_C^+^ with dimethyl sulfide, benzene, and indole (Figs S14, S15, Table S12). These model calculations suggest that the interactions between carbocations and sulfur are comparable to those of carbocations and π systems. For all model carbocations, the carbocation is covalently bonded to the sulfur atom via a dative bond. In the case of benzene and indole, the interaction with CH_3_^+^, CH_3_CH_2_^+^, and (CH_3_)_2_CH^+^ are covalent, while for (CH_3_)_3_C^+^ the interaction with the π system is noncovalent.

Of particular interest is a comparison of noncovalent interaction between (CH_3_)_3_C^+^ and dimethyl sulfide, benzene, and indole, as these are likely to be most relevant for TPSs. To this end we performed a constrained minimization between (CH_3_)_3_C^+^ and dimethyl sulfide to mimic a situation where steric constraints preclude dative bond formation (Tables S11, S12). The gas‐phase interaction energies for (CH_3_)_3_C^+^ with dimethyl sulfide, benzene, and indole are −12.4, −13.8, and −20.6 kcal·mol^−1^, respectively. A SAPT2 break‐down of the energy contribution toward this interaction shows that for both sulfur–cation and π–cation the interactions are a combination of electrostatics, induction, and dispersion (Table S13).

In summary, our EnzyDock QM/MM simulations provide structural and energetic support for the catalytic roles of M188 and M304 in stabilizing carbocation intermediates through sulfur–cation interactions, corroborating insights from our current structural and previous mutagenesis studies. Specifically, M188 is critical for catalysis by stabilizing the early intermediates B and B′. Additionally, our results suggest that the combined mutations of M75 and M304 can modulate product selectivity: while M75 promotes the formation of both IE A and IE B, M304 appears to stabilize a branching‐point carbocation intermediate that directs the reaction toward either IE A or IE B (Scheme S1). This hypothesis is examined by mutagenesis studies, which are described below.

### Variant design for selective IE A/IE B production

**Fig. 5:**
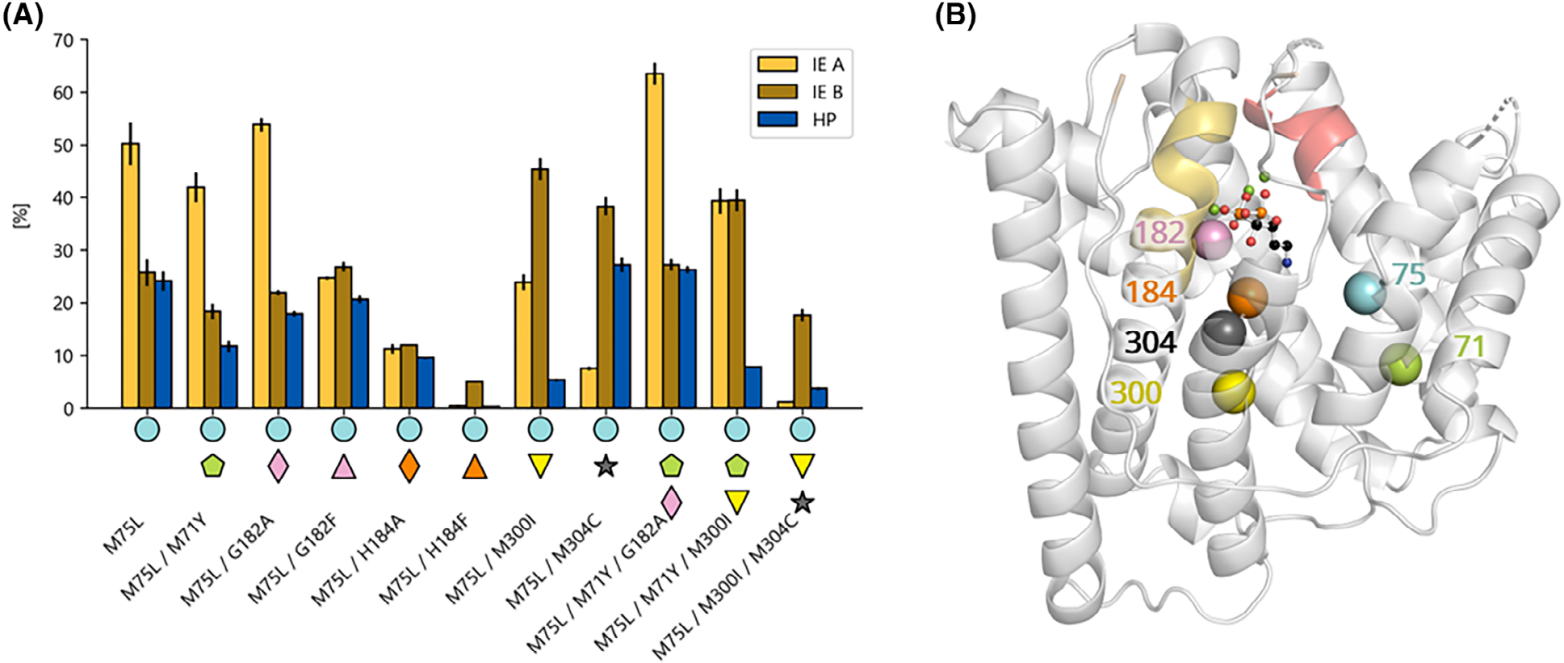
Analysis of HpS variants with respect to terpene product profile. It is displayed as percental ratio of the areas of the respective GC–FID product peaks; error bars represent the mean values ± standard deviation over triplicates. Values are normalized with the HpS^M75L^ yield set as 100%. (A) Respective relative product profiles of HpS double and triple variants in respect to HpS^M75L^. HpS variants displayed as colored symbols: M75L cyan circle, M71Y green pentagon, G182A pink diamond, G182F pink triangle, H184A orange diamond, M300I yellow triangle, M304C gray star. Respective spectra are shown in Figs S6, S9. (B) The structure of HpS^M75L^·Mg^2+^_3_·AHD is shown in cartoon representation. Location of HpS variants are displayed as colored spheres. The alendronate (AHD) molecule is shown as black sticks. Same view as in Fig. 1C.

To further explore how modifications within the active site affect catalysis, we conducted additional mutational studies aimed at enhancing IE A production and gaining deeper insight into the HpS reaction cascade [15]. In the first step, one or two additional mutations were introduced in the HpS^M75L^ variant, primarily targeting residues within the methionine‐rich motif and others implicated by structural modeling to influence the reaction mechanism. We focused especially on positions, such as G182, whose side chain points away from the active site but may influence the backbone conformation of the catalytically relevant G183. and H184, which may act as an active‐site base. Based on these criteria, we constructed a series of double variants: HpS^M75L/M71Y^, HpS^M75L/G182A^, HpS^M75L/G182F^, HpS^M75L/H184A^, HpS^M75L/H184F^, HpS^M75L/M300I^, and HpS^M75L/M304C^. All variants were expressed in *E. coli*, and their product profiles were analyzed in comparison with HpS^M75L^ (Fig. 5A, Figs S4–S7). Among them, HpS^M75L/M71Y^ and HpS^M75L/G182A^ exhibited terpene profiles similar to the original M75L variant. Notably, HpS^M75L/G182A^ maintained a comparable total terpene shield (~93.6%) but achieved the highest IE A content (~54%). In contrast, HpS^M75L/M71Y^ produced a reduced total terpene yield (~72%) and a lower proportion of IE A (~42%).

The variants HpS^M75L/G182F^, HpS^M75L/M300I^, and HpS^M75L/M304C^ all show total terpene yields ranging from 72% to 75% relative to HpS^M75L^. However, their product distributions differ markedly—not only from HpS^M75L/M71Y^ but also from each other. In all three cases, IE B emerges as the most abundant product. For HpS^M75L/G182F^, the major products—IE A, IE B, and HP—are formed in relatively similar amounts (~25%, ~27%, and ~ 21%, respectively). In contrast, HpS^M75L/M300I^ produces the highest yield of IE B among all tested HpS variants (~45%), followed closely by HpS^M75L/M304C^ (~38%). Interestingly, these two variants differ substantially in their second most abundant product. HpS^M75L/M300I^ generates ~24% IE A and only ~5% HP, whereas HpS^M75L/M304C^ shows the opposite trend, producing ~7% IE A and a significantly higher amount of HP (~27%). This latter finding aligns with our EnzyDock QM/MM simulations described above, which suggested that M304 plays a key role in modulating the reaction pathway between IE A and IE B.

In HpS^M75L/H184A^ and in HpS^M75L/H184F^ the total terpenoid production is significantly reduced (~33% and ~6%; Fig. 5A, Fig. S5a and Table S4). In the HpS^M75L/H184A^ variant, as observed for HpS^M75L/G182F^, the products IE A (~11%), IE B (~12%), and HP (~10%) are formed in similar proportions, with a slight preference for IE B. By contrast, HpS^M75L/H184F^ primarily produces IE B (~5%), with negligible amounts of the other products. These results suggest that combining the M75L background with H184 mutations primarily impacts the enzyme's overall reactivity, but does not lead to a more favorable or selective product distribution.

To summarize the effects of the double variants, we focus on the most distinct findings. The exchange of the small glycine at position 182 for a hydrophobic alanine adds little compared with the effect of the single M75L mutation. When replacing the glycine by a larger hydrophobic side chain (*i.e*., phenylalanine) the preference for IE A production is lost. Thus, a leucine at position 75 in combination with alanine at position 182 may facilitate either the stabilization of carbocations in the early stages of the catalytic cycle (which is essential for IE forming pathways) or a more rapid GGDP to GLDP isomerization, thereby favoring IE A production. However, the exact mechanism for discrimination between IE A and IE B on a molecular level still remains obscure. The preference of HpS^M300I^ and HpS^M304C^ for IE B may indicate a more rapid deprotonation of D′ by PP_i_ due to reorientation of this intermediate. Interestingly, we did not detect any formation of HPol within these double variants. Thus, we conclude that the introduced mutations are potentially hindering the incorporation of a water moiety to ultimately form HPol.

Since double variants HpS^M75L/G182A^, HpS^M75L/M300I^ and HpS^M75L/M304C^ showed promise as catalysts for the optimized production of IE A or IE B, respectively, further improvements in yield and product specificity were attempted by generating triple variants HpS^M75L/M71Y/G182A^, HpS^M75L/M71Y/M300I^, and HpS^M75L/M300I/M304C^ (Fig. 5, Figs S8, S9, and Table S5). HpS^M75L/M71Y/G182A^ was expected to raise the portion of IE A while maintaining low IE B and HP production. HpS^M75L/M71Y/M300I^ was generated to lower the amount of HP production and finally HpS^M75L/M300I/M304C^ was chosen to test the shift of the product profile toward IE B as main product. Variant HpS^M75L/M71Y/G182A^ demonstrated excellent IE A production (~64% yield) as well as increased relative total terpene yield compared with HpS^M75L^. The combination of the IE A‐favoring M71Y variant and IE B‐enhancing M300I resulted in equal production of both isoelisabethatriene isoforms (~39% each) but decreased relative total terpene yield (~86%). Interestingly, the combination of the M300I and M304C mutations was strongly detrimental to the total terpene production but did create a triple variant with almost exclusive IE B production (Fig. 5, Figs S5, S9 and Table S5).

### Bioinformatic and structural evidence for conserved methionine motifs

Structure‐ and mechanism‐guided mutagenesis of HpS revealed that Met residues within the active site are key modulators of product selectivity between IE A and IE B. By introducing targeted mutations, particularly at M75, M300, and M304, we uncovered how sulfur–cation interactions and steric effects influence the stabilization of carbocation intermediates and shift the product profile. These insights enabled the design of double and triple variants with enhanced or selective production of valuable diterpene scaffolds, advancing the enzymatic engineering of sustainable bioactive compounds. The proposed role of Met residues in HpS raises the question of a more general role for methionine residues in TPS. To address this question, we extracted Class I TPS from the Protein Data Bank (PDB) and analyzed their active sites. The PDB has 64 plant and 92 microbial Class I TPS, and of these, 21 are unique plant and 40 are unique microbial TPS. We enumerated the number of Met residues in the active site, which range from 0–3. The most common number of Met residues in the active site are 0–2, while 3 Met residues was only observed for one case. Hence, we conclude that the case of HpS, which has 5 Met residues in the active site is a rather unusual case.

## Conclusion

Our study uncovers a novel catalytic role for methionine residues in terpene synthases, providing the first indirect experimental evidence for sulfur–carbocation stabilization in this enzyme class. Through a combination of crystal structure determination, multiscale QM/MM EnzyDock simulations, model quantum chemistry calculations, and targeted mutagenesis, we demonstrate that M188 and M304 in HpS from *S. clavuligerus* directly contribute to the stabilization of key carbocation intermediates via sulfur–cation interactions. This interaction type, though previously hypothesized from computational studies, had not yet been experimentally validated via mutational studies in terpene synthases.

In particular, mutation of M188 resulted in complete enzymatic inactivation, supporting its critical role in stabilizing the early‐stage cyclization product. M304 was also identified as catalytically relevant, while M300, which is spatially adjacent to several aromatic residues, likely plays a structural rather than catalytic role through sulfur–π interactions. Beyond these specific residues, our findings suggest that methionine‐rich active‐site motifs may represent a previously unrecognized strategy in terpene synthases for guiding highly reactive carbocation intermediates through complex reaction cascades. A bioinformatic survey revealed 333 related TPS sequences featuring methionine residues aligned with M75, suggesting potential conservation of this catalytic feature. However, the diversity of sequence contexts implies that this adaptation may support specific terpene scaffolds rather than a universal TPS mechanism.

Model quantum chemical calculations demonstrate that methionine‐carbocation dative bonds can be highly potent (ca. −25 to −30 kcal·mol^−1^), although explicit QM/MM optimizations suggest that active‐site steric congestion precludes such coordination in the enzyme, favoring strong noncovalent stabilization instead. SAPT2 analysis further reveals that these sulfur–cation interactions are energetically comparable to traditional π–cation interactions, driven by a combination of electrostatic, inductive, and dispersive forces. These findings underscore a distinctive mechanistic motif in HpS where methionine‐rich environments can provide noncovalent electronic stabilization essential for steering complex terpene cyclizations.

We also leveraged mechanistic insights to rationally engineer HpS variants with enhanced selectivity for the pseudopterosin precursors IE A and IE B. Triple variants achieved highly selective product profiles, establishing HpS as a customizable platform for sustainable biosynthesis of high‐value diterpenoids with pharmaceutical and cosmetic applications. Finally, our work supports a broader hypothesis: Met residues may serve as versatile stabilizing elements for carbocations across diverse enzyme families, including aromatic prenyltransferases (APTs), where similar active‐site configurations suggest parallel sulfur–cation interactions [68]. In particular, the active‐site methionine in APT NphB (PDB ID: 1ZB6 [69]) is well‐positioned to engage with prenyl carbocations, further suggesting that the catalytic role of methionine could be underappreciated and widespread in enzymatic carbocation chemistry.

## Conflict of interest

The authors declare that the research was conducted in the absence of any commercial or financial relationships that could be construed as a potential conflict of interest.

## Author contributions

TB, DTM, and BL conceived the study. CPOH and RD performed the protein expression, purification, crystallization, diffraction data collection, and refinement. RD, CPOH, and BL analyzed the structural data. MRi and MRe performed cloning, activity assays, and analyzed the activity assays with guidance from TB and DG, MRi and EB performed the cloning, cultivation experiments, and activity assays. SZ, RS, and DTM performed the enzyme calculations. GF and MB performed the sequence search. GS, BL, DTM, and TB supervised the project. All authors wrote the manuscript.

## Supporting information


**Fig. S1.** Polder electron density map.
**Fig. S2.** Characterization of HpS^M75L^.
**Fig. S3.** Primary sequence of HpS with secondary structure elements.
**Fig. S4.** Growth curves of the expression of HpS double variants.
**Fig. S5.** Analysis of HpS variants with respect to total terpene yield.
**Fig. S6.** GC/MS analysis of different HpS double variants.
**Fig. S7.** GC–MS spectra of target compounds.
**Fig. S8.** Growth curves of the expression of HpS triple variants.
**Fig. S9.** GC/MS analysis of different HpS triple variants.
**Fig. S10.** Selected reaction states along the pathways to HP and HPol.
**Fig. S11.** Selected reaction states along the pathways to IE A and IE B.
**Fig. S12.** Superposition of the final carbocations forming HP/HPol.
**Fig. S13.** Energy profiles in the gas phase.
**Fig. S14.** Model calculations of the complexation energy.
**Fig. S15.** Illustrative examples of parallel interactions.
**Scheme S1.** Suggested mechanism for hydropyrene, hyropyrenol, isoelisabethatriene A/B.
**Table S1.** Data collection, refinement, and validation statistics.
**Table S2.** Modell completeness given for each protein chain.
**Table S3.** Results of a DALI search.
**Table S4.** Relative proportions of IE A, IE B and HP of HpS double ariant.
**Table S5.** Relative proportions of IE A, IE B and HP of HpS triple variants.
**Table S6.** Values used to filter Interpro name column.
**Table S7.** Nuclear Overhauser effect (NOE) restraints for EnzyDock docking.
**Table S8.** Oligonucleotides used for cloning.
**Table S9.** QM(M06‐2X)/MM energies leading to HP and HPol.
**Table S10.** QM(M06‐2X)/MM energies leading to IE A and IE B.
**Table S11.** Model calculations of the complexation energy between DMS and cation B or B′.
**Table S12.** Model calculations of the complexation energy between fragments benzene, indole, and DMF.
**Table S13.** Model SAPT2 noncovalent interaction energy.

**Movie S1.** Movie of HpS^M75L^ overall fold and active site.

## Data Availability

The authors declare that all data supporting the findings of this study are available within the article and its Supplementary Information Files or from the corresponding author upon reasonable request. The atomic coordinates have been deposited in the Protein Data Bank with the accession code 8B4L (Se‐Met HpS^M75L^·Mg^2+^_3_·AHD) and 8B4M (HpS^M75L^·Mg^2+^_3_·AHD).
