# Supplementary material for: A methionine‐lined active site governs carbocation stabilization and product specificity in a bacterial terpene synthase
Source: FEBS Lett. 2026 Mar 19;600(11):1621–37. doi: 10.1002/1873-3468.70325 (PMC13244428; doi:10.1002/1873-3468.70325)
Supplement: Supplementary file 1 — Fig. S1. Polder electron density map. Fig. S2. Characterization of HpSM75L. Fig. S3. Primary sequence of HpS with secondary structure elements. Fig. S4. Growth curves of the expression of HpS double variants. Fig. S5. Analysis of HpS variants with respect to total terpene yield. Fig. S6. GC/MS analysis of different HpS double variants. Fig. S7. GC–MS spectra of target compounds. Fig. S8. Growth curves of the expression of HpS triple variants. Fig. S9. GC/MS analysis of different HpS triple variants. Fig. S10. Selected reaction states along the pathways to HP and HPol. Fig. S11. Selected reaction states along the pathways to IE A and IE B. Fig. S12. Superposition of the final carbocations forming HP/HPol. Fig. S13. Energy profiles in the gas phase. Fig. S14. Model calculations of the complexation energy. Fig. S15. Illustrative examples of parallel interactions. Scheme S1. Suggested mechanism for hydropyrene, hyropyrenol, isoelisabethatriene A/B. Table S1. Data collection, refinement, and validation statistics. Table S2. Modell completeness given for each protein chain. Table S3. Results of a DALI search. Table S4. Relative proportions of IE A, IE B and HP of HpS double ariant. Table S5. Relative proportions of IE A, IE B and HP of HpS triple variants. Table S6. Values used to filter Interpro name column. Table S7. Nuclear Overhauser effect (NOE) restraints for EnzyDock docking. Table S8. Oligonucleotides used for cloning. Table S9. QM(M06‐2X)/MM energies leading to HP and HPol. Table S10. QM(M06‐2X)/MM energies leading to IE A and IE B. Table S11. Model calculations of the complexation energy between DMS and cation B or B′. Table S12. Model calculations of the complexation energy between fragments benzene, indole, and DMF. Table S13. Model SAPT2 noncovalent interaction energy. [file FEB2-600-1621-s001.docx]

**Supporting information**

A Methionine-lined active site governs carbocation stabilization and product specificity in a bacterial terpene synthase

Marion Ringel^1,⊥^, Carl P. O. Helmer^2,⊥^, Shani Zev^3,⊥^, Ronja Driller^2^, Emily Buhr^1^, Markus Reinbold^1^, Renana Schwartz^3^, Gabriel Foley^4^, Mikael Boden^4^, Daniel Garbe^1^, Gerhard Schenk^4,5,6^, Dan Thomas Major^3,*^, Bernhard Loll^2,*^, and Thomas Brück^1,*^

^1^ Werner Siemens-Chair of Synthetic Biotechnology, Technical University of Munich (TUM), TUM School of Natural Sciences; Garching, 85748, Germany.

^2^ Institute of Chemistry and Biochemistry, Laboratory of Structural Biochemistry, Freie Universität Berlin; Berlin, 14195, Germany.

^3^ Department of Chemistry and Institute for Nanotechnology & Advanced Materials, Bar-Ilan University; Ramat-Gan, 52900, Israel.

^4^ School of Chemistry and Molecular Biosciences, The University of Queensland; Brisbane, Queensland 4072, Australia.

^5^ Sustainable Minerals Institute, The University of Queensland; Brisbane, Queensland 4072, Australia.

^6^ Australian Institute of Bioengineering and Nanotechnology, The University of Queensland; Brisbane, Queensland 4072, Australia.

^⊥^ M.R., C.P.O.H. and S.Z. contributed equally

Corresponding authors

* Thomas Brück - Werner Siemens-Chair of Synthetic Biotechnology, Technical University of Munich (TUM), TUM School of Natural Sciences; Garching, 85748, Germany.

* Bernhard Loll - Institute of Chemistry and Biochemistry, Laboratory of Structural Biochemistry, Freie Universität Berlin; Berlin, 14195, Germany.

* Dan Thomas Major - Department of Chemistry and Institute for Nanotechnology & Advanced Materials, Bar-Ilan University; Ramat-Gan, 52900, Israel.

**Supplementary Text**

**Structure determination.**

Initially, HpS^WT^ crystals diffracted to only 15 Å. To improve diffraction quality, we tested different affinity tags fused to HpS, as well as HpS variants, in absence or presence of the bisphosphonate inhibitor alendronate (AHD). AHD mimics the TPS substrate GGDP and can therefore aid in obtaining the closed conformation of HpS. Better crystals were obtained for HpS^M75L^ diffracting to a resolution limit of 3.04 Å. To solve the phasing, HpS^M75L^ was labelled with selenomethionine (SeMet-HpS^M75L^) and subsequently co-crystallized with AHD (SeMet-HpS^M75L^•Mg^2+^_3_•AHD). The structure was solved by single anomalous dispersion (**Supplementary Table S1**). Phase determination was complicated due to a weak anomalous signal and strong translational non-crystallographic symmetry. However, we could locate 56 selenium sites within the asymmetric unit corresponding to six molecules of SeMet-HpS^M75L^. The unlabelled HpS^M75L^ incubated with AHD (HpS^M75L^•Mg^2+^_3_•AHD) crystallized in a different space group than SeMet-HpS^M75L^•Mg^2+^_3_•AHD, and the crystals diffracted to higher resolution (**Supplementary Table S1**). The structure of HpS^M75L^•Mg^2+^_3_•AHD was solved by molecular replacement, utilizing the structure of SeMet-HpS^M75L^, and given its higher resolution, it then also enabled us to further complete the model of HpS^M75L^. Improvements of the structural models for both SeMet-HpS^M75L^•Mg^2+^_3_•AHD and HpS^M75L^•Mg^2+^_3_•AHD were achieved by switching between the two data sets for completing and correcting each model. Overall, the amino acid sequence assignment was greatly supported by the anomalous signals from the selenium sites.

**Overall structure.**

Due to flexibility, the electron density for the last five C-terminal residues is ill defined and did not allow any detailed interpretation. Furthermore, at a resolution of 3.04 Å it was not possible to clearly resolve the exact position and hence the coordination of the three Mg^2+^ ions as well as the AHD molecule. Therefore, we positioned the metal ions and AHD in the electron density based on structural similarity to other TPSs with bound Mg^2+^ and AHD (**Supplementary Table S2,** and **Supplementary Fig. S4,** and **Supplementary Movie S1**). Notably, there are differences in the quality of the electron density of the six polypeptides in the asymmetric unit due to protein packing. Consequently, in the structure of HPS^M75L^•Mg^2+^_3_•AHD we could merely model four out of six active sites with Mg^2+^ and AHD present. The different monomers might also represent a mixture of fully and partially closed active site conformations, an interpretation that is supported by the observation that in monomers with bound Mg^2+^ and AHD the electron density of the C-terminal residues is better defined, thus enabling model building; **Supplementary Table S3**). A similar observation has been reported for the bacterial diterpene synthase CotB2 (PDB-ID 6GGI and 6GGJ),[1] where upon binding of the substrate, the C-terminus folds over the active site.[1]

**
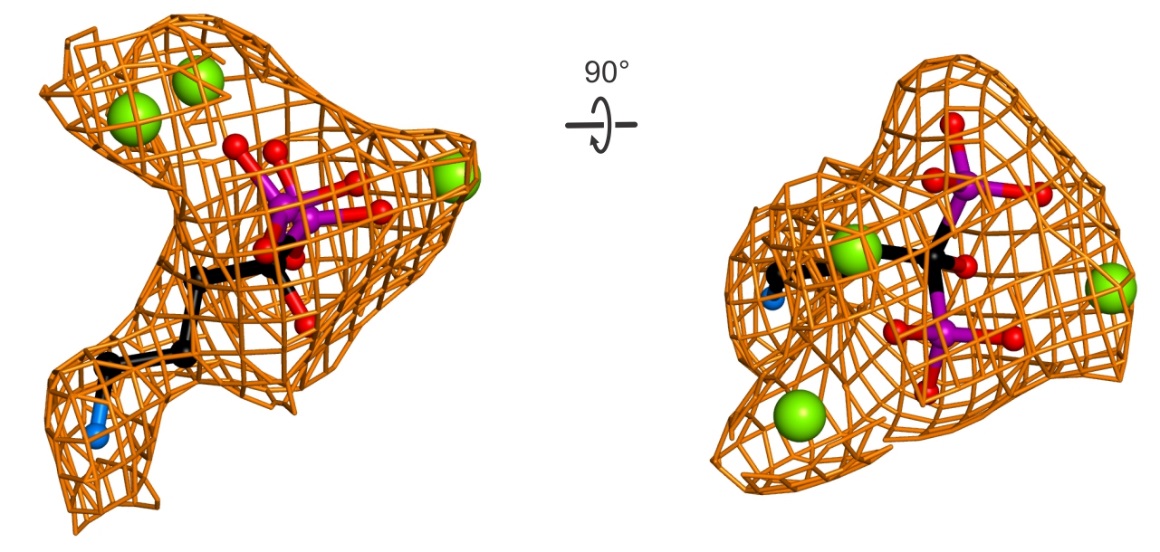
**

**Supplementary Fig. S1** Same view as in Fig. 1c. Polder electron density map[2] around AHD molecule and Mg^2+^ ions shown as mesh at a σ-level of 3.0. The AHD molecule is presented as ball-stick-model with carbon atoms colored in black, oxygen in red, phosphorous in purple and nitrogen in blue. Mg^2+^ ions are shown as green spheres.

**
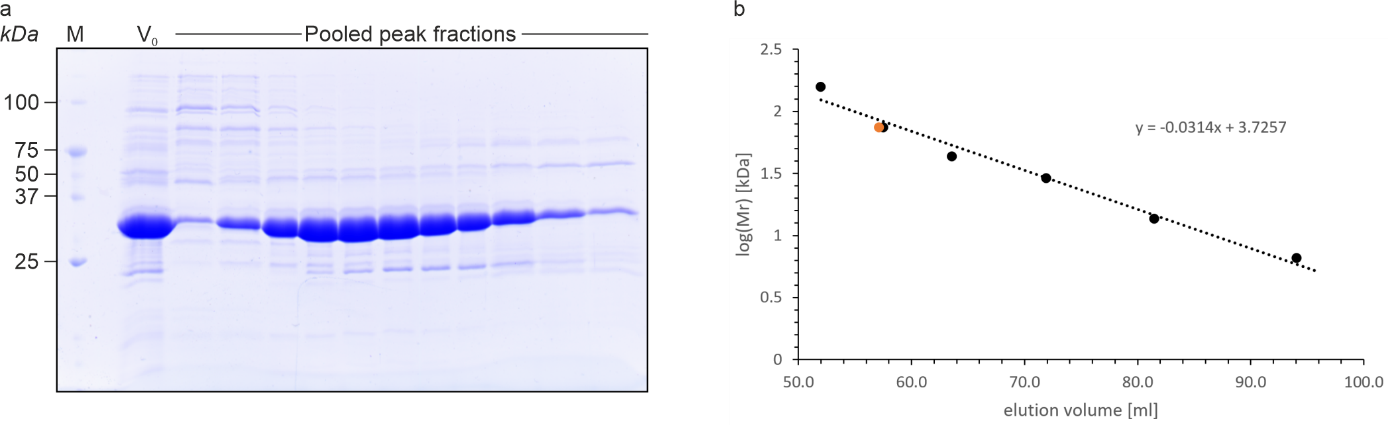
**

**Supplementary Fig. S2** Characterization of HpS^M75L^. (a) SDS-PAGE corresponding to the final size exclusion chromatography run. M: Marker. V_0_: void peak. (b) Calibration curve of Superose S75 16/60. Proteins used for the calibration curve are: Aldolase (158.0 kDa), Conalbumin (75.0 kDa), Ovalbumin (43.0 kDa), Carbonic anhydrase (29.0 kDa), Ribonuclease A (13.7 kDa), and Aprotinin (6.5 kDa). Dimeric HpS^M75L^ elutes at a volume of 57.1 ml (orange filled circle) corresponding to a molecular mass of 74 kDa.

**
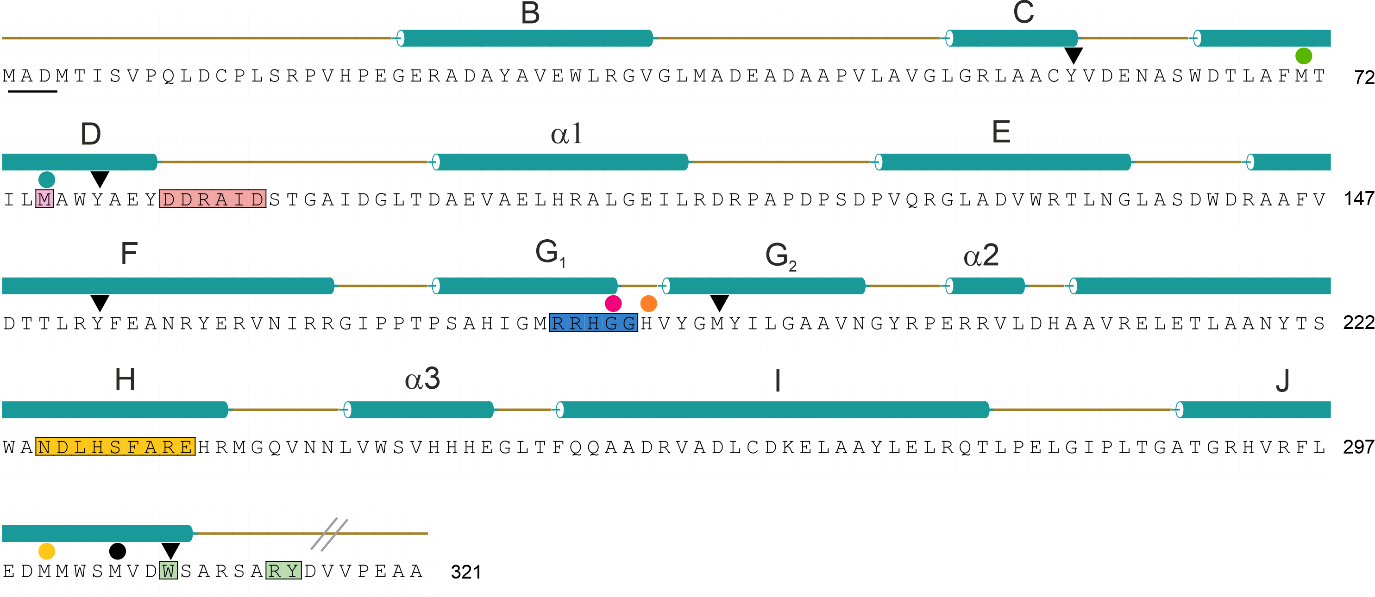
**

**Supplementary Fig. S3** On top of the primary sequence of HpS the secondary structure elements are drawn. The aspartate-rich motif is highlighted in red and the NSE motif in yellow. The effector motif is colored in blue. The RY-dimer as well as the upstream tryptophan are highlighted in light green. The location of the M75L exchange is highlighted by a pink square. Residues subjected to mutagenesis are colored as filled spheres in Fig. 5: M71 (green), M75 (cyan), G182 (magenta), H184 (orange), M300 (yellow), and M304 (black). Black arrows indicate aromatic amino acids (Y58, Y78, H184, M188, Y153, and W307) pointing into the active site.





**Supplementary Fig. S4** Growth curves of the expression of HpS double variants at 22 °C for 48 h in shaking flasks. Error bars represent the mean values ± standard deviation over biological triplicates. (a) The growth curves of the following HpS^M75L^ (red) double variants are shown: HpS^M75L/G182A^ (blue), HpS^M75L/G182F^ (green) and HpS^M75L/H184A^ (orange). (b) The growth curves of the following HpS^M75L^ (orange) double variants are shown: HpS^M75L/M71Y^ (red), HpS^M75L/M304C^ (black), HpS^M75L/M300I^ (green) and HpS^M75L/H184F^ (blue). OD_600_ was measured at defined time points (dots show the different measurement points). The growth curves were fitted by a sigmoidal fit.


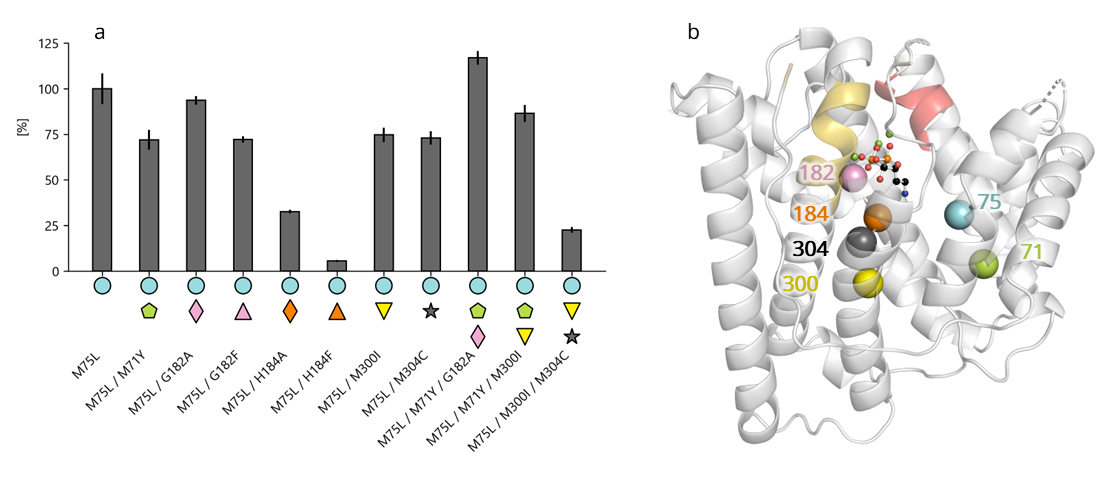
 **Supplementary Fig. S5** Analysis of HpS variants with respect to total terpene yield, displayed as percentage ratio of the areas of the respective GC-FID product peaks, total terpene yield is displayed relative to HpS^M75L^. Error bars represent the mean values ± standard deviation over triplicates. (a) HpS variants displayed as colored shapes. Each color represents a different mutation site, and each shape represents the resulting amino acid after mutation: M75L as cyan-colored sphere, M71Y as green pentagon, G182F as pink diamond, G128F as pink triangle, H184A as orange diamond, M300I as yellow upside triangle, M304C as black star. (b) The structure of HpS^M75L^•Mg^2+^_3_•AHD is shown in cartoon representation. Location of HpS variants are displayed as colored spheres (see color code above). The AHD molecule is shown in stick representation. Same view as in Fig. 1b.


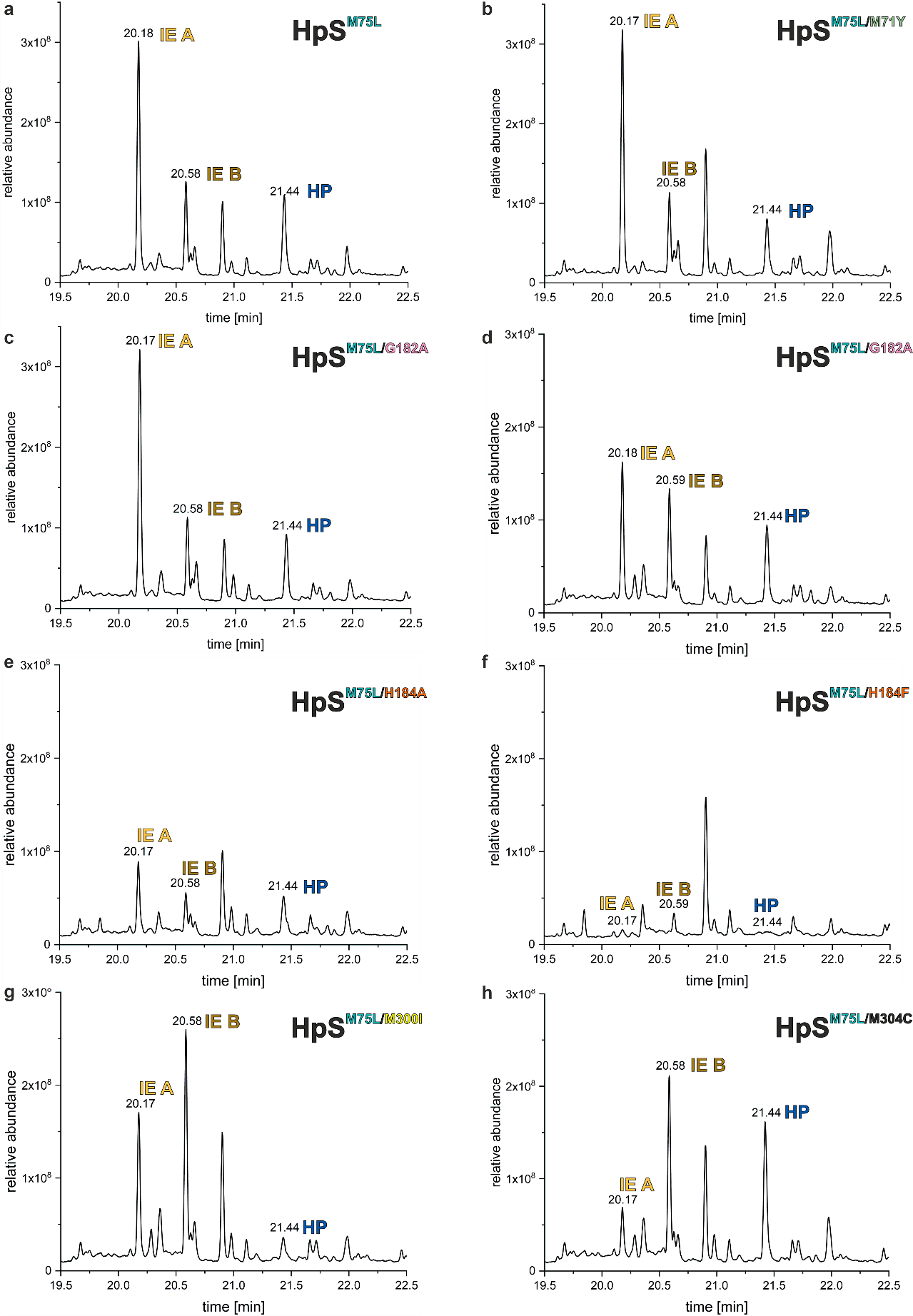


**Supplementary Fig. S6** Product profiles of different HpS double variants as shown in GC-MS chromatograms with respect to the main products isoelisabethatriene A (RT = 20.17 min), isoelisabethatriene B (RT = 20.59 min) and hydropyrene (RT = 21.44 min). Qualitative GC-MS spectra are presented in Supplementary Fig. 7. (a) HpS^M75L^ (b) HpS^M75L/M71Y^ (c) HpS^M75L/G182A^ (d) HpS^M75L/G182F^ (e) HpS^M75L/H184A^ (f) HpS^M75L/H184F^ (g) HpS^M75L/M300I^ (h) HpS^M75L/M304C^.


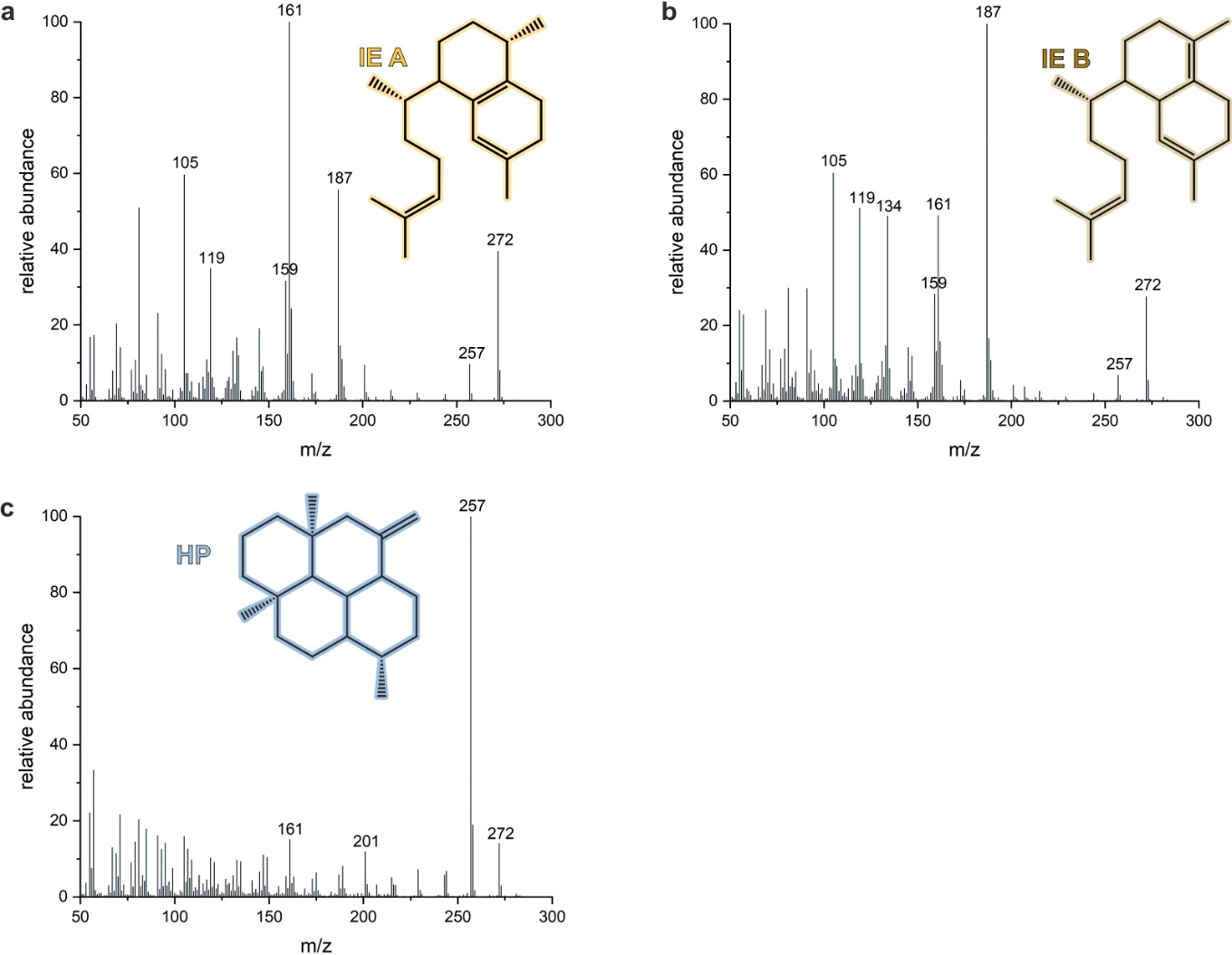


**Supplementary Fig. S7** GC-MS spectra of target compounds (a) isoelisabethatriene A, (b) isoelisabethatriene B (c) hydropyrene.





**Supplementary Fig. S8** Growth curves of the expression of HpS triple variants at 22 °C for 48 h in shaking flasks. Error bars represent the mean values ± standard deviation over biological triplicates. The cell densities (OD_600_) were measured at defined time points (filled circles show the different measurement points). The expressed HpS^M75L^ (green) triple variants are HpS^M75L/M71Y/G182A^ (black), HpS^M75L/M300I/M304C^ (blue) and HpS^M75L/M71Y/M300I^ (red). The curves were fitted by a sigmoidal fit.

**
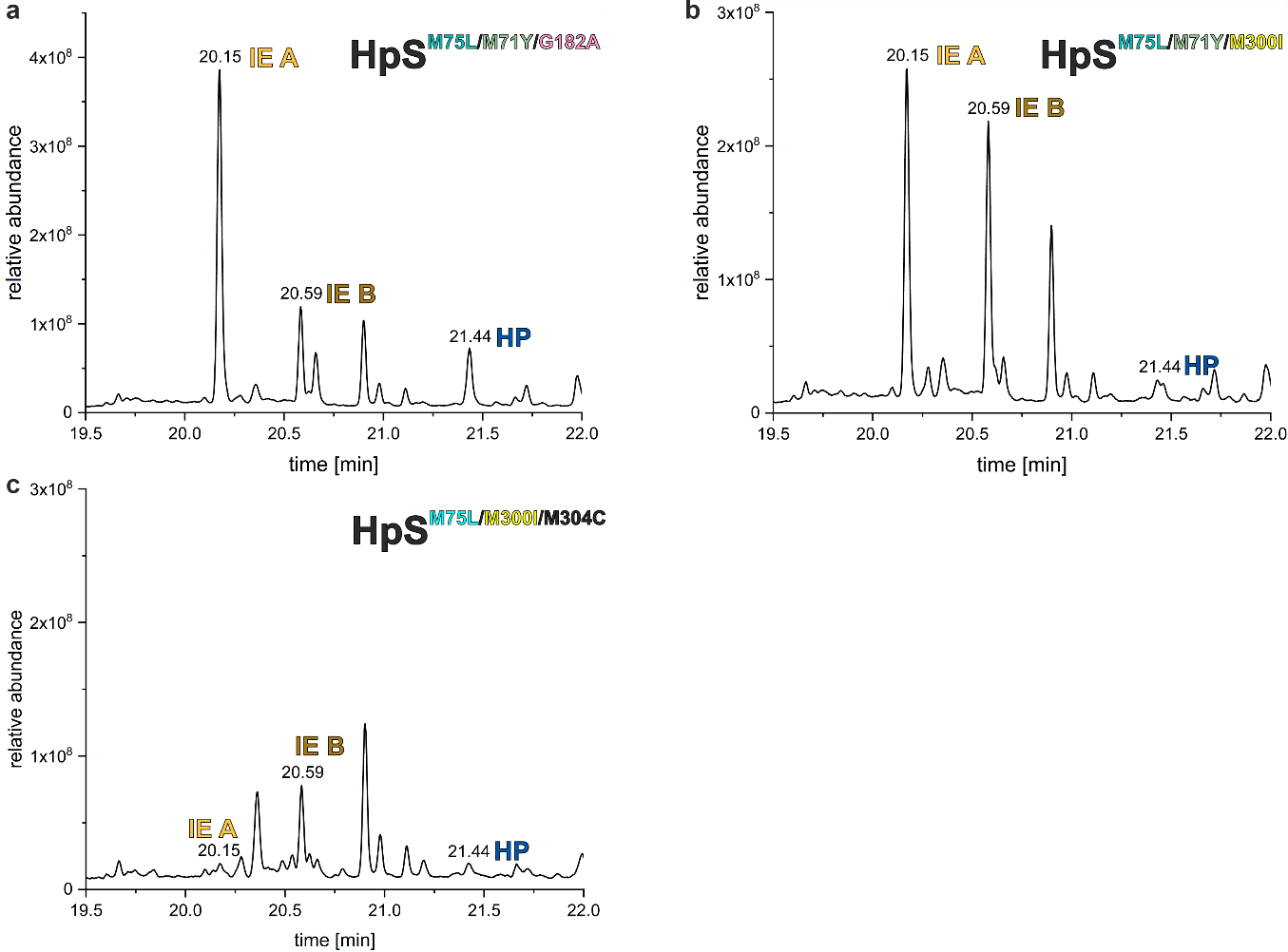
**

**Supplementary Fig. S9** Product profiles of different HpS triple variants as shown in GC-MS chromatograms with respect to the main products isoelisabethatriene A (RT = 20.17 min), isoelisabethatriene B (RT = 20.59 min) and hydropyrene (RT = 21.44 min). (a) HpS^M75L/M71Y/G182A^ (b) HpS^M75L/M71Y/M300I^ (c) HpS^M75L/M300I/M304C^. Qualitative GC-MS spectra of the respective compounds are presented in Fig. S7.

**Supplementary Fig. S10** Selected reaction states along the pathways to HP and HPol docked into HpS^WT^ using EnzyDock with the substrate GGDP and subsequent carbocations **B**, **C**, **D**, **F,** and **I**. The different docked intermediates are shown in grey, the PP_i_ group in orange and three Mg^2+^ ions in green. Key residues of HpS^WT^ are highlighted.

**Supplementary Fig.S11** Selected reaction states along the pathways to IE A and IE B docked into HpS^M75L^ using EnzyDock with substrate GGDP, GLDP and carbocations **B’**, **C’**, **D’** and **E’**. The different docked intermediates are shown in grey, the PP_i_ group in orange and three Mg^2+^ ions in green. Key residues of HpS^M75L^ are highlighted.


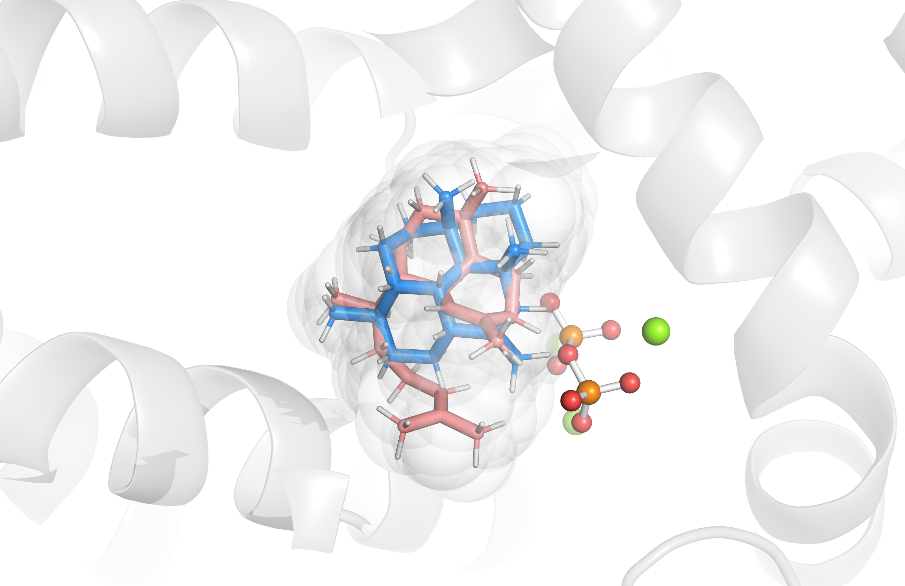


**Supplementary Fig. S12** Superposition of the final carbocations forming HP/HPol (blue) or IE A/B (red). Green spheres indicate the positions of Mg^2+^ ions.


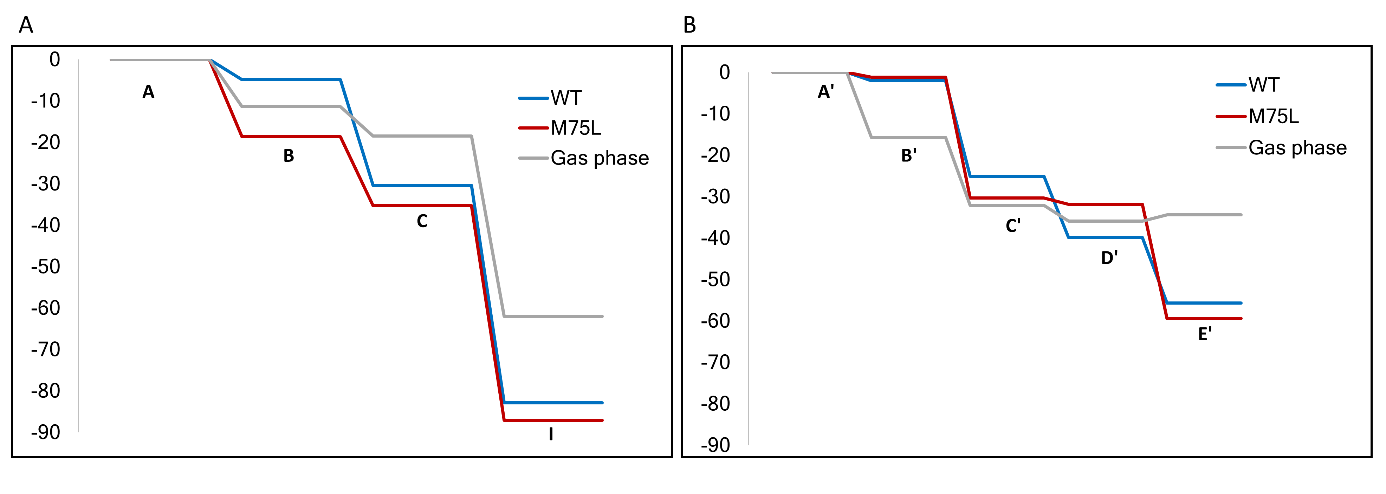


a

b

**Supplementary Fig. S13** (a) Energy profile for the formation of hydropyrene cation **I** in the gas phase, HpS^WT^, and HpS^M75L^. (b) Energy profile for the formation of IE cation **E’** in the gas phase, HpS^WT^, and HpS^M75L^.


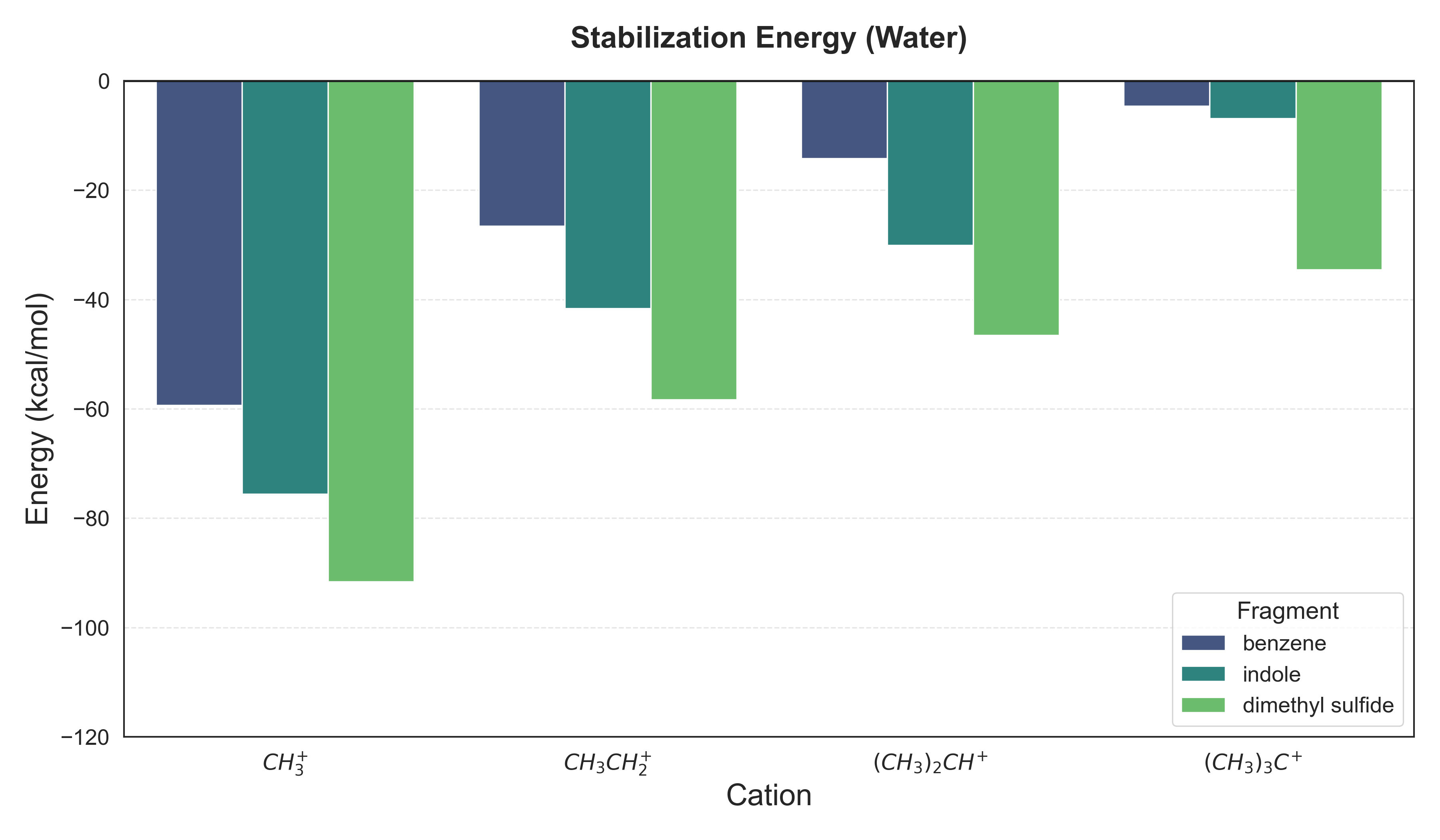

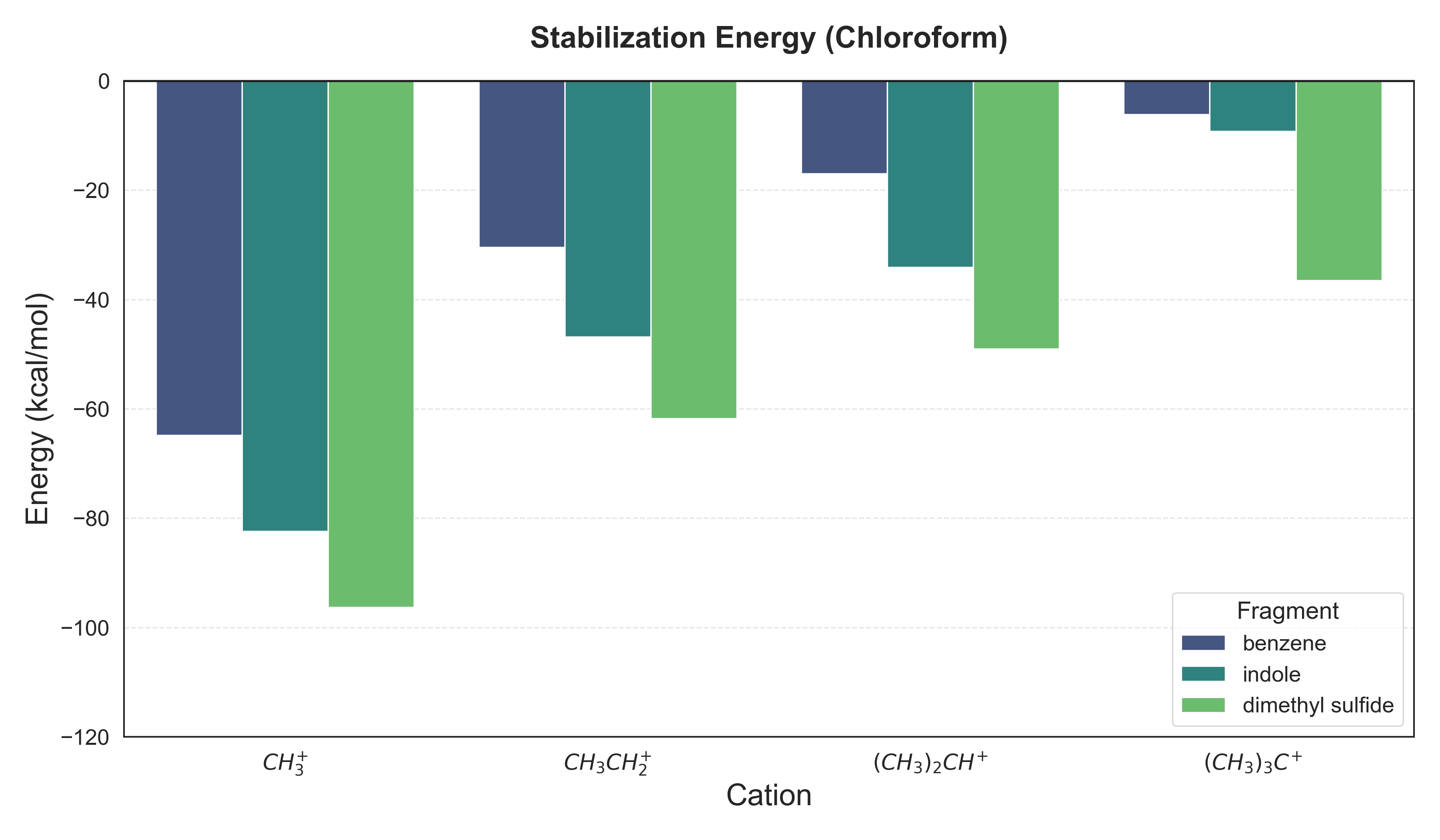

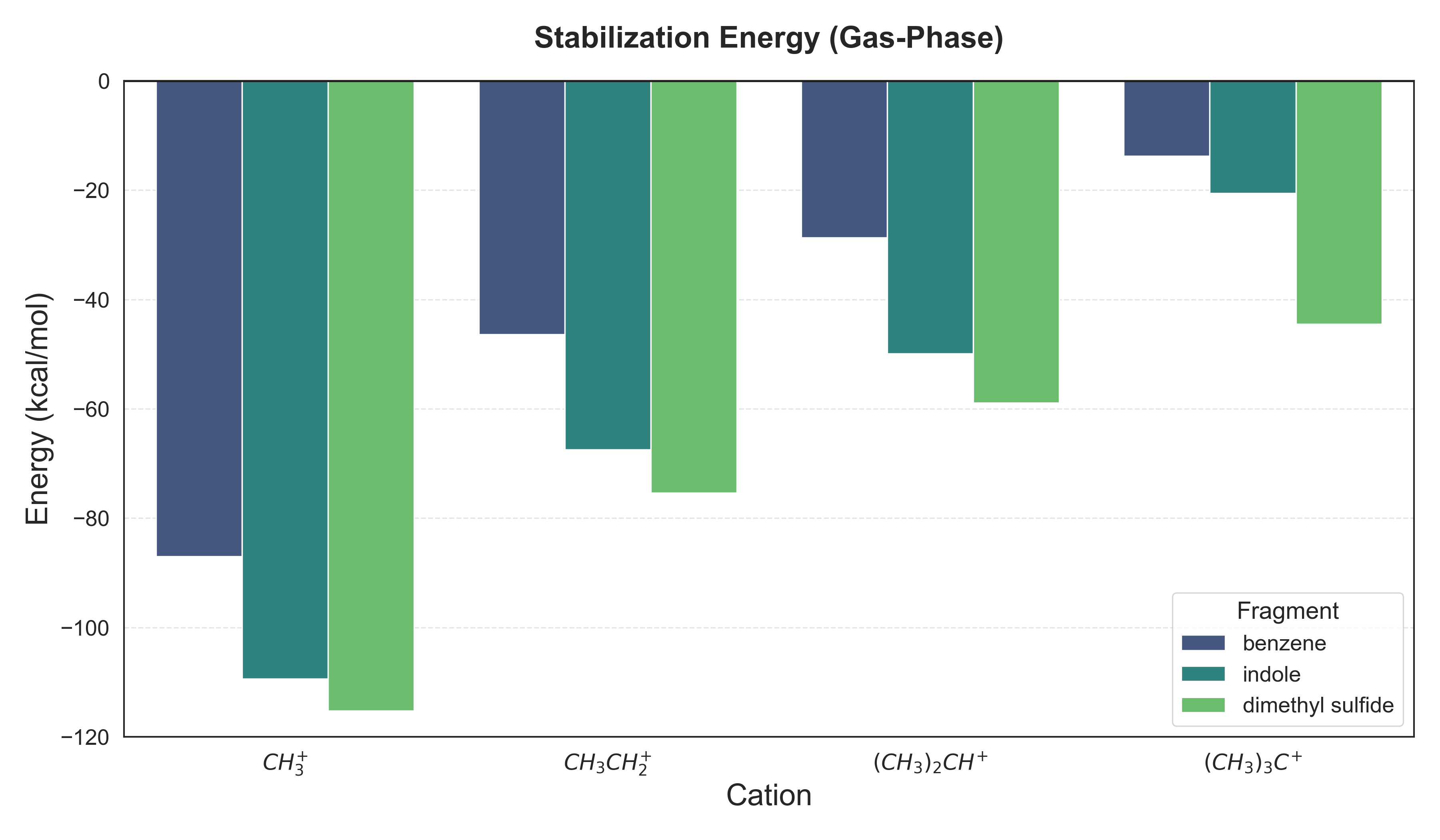


**Supplementary Fig. S14** Model calculations of the complexation energy (kcal/mol) between fragments benzene, indole, and dimethyl sulfide with model cations in gas-phase, chloroform, and water (parallel orientation). The complexation energy was calculated as Δ*E = E^C-F^ –* (*E^C^ + E^F^*) where C is the model cation and F the fragment. Method: $\omega$B97M-V/def2-TZVPD.

**
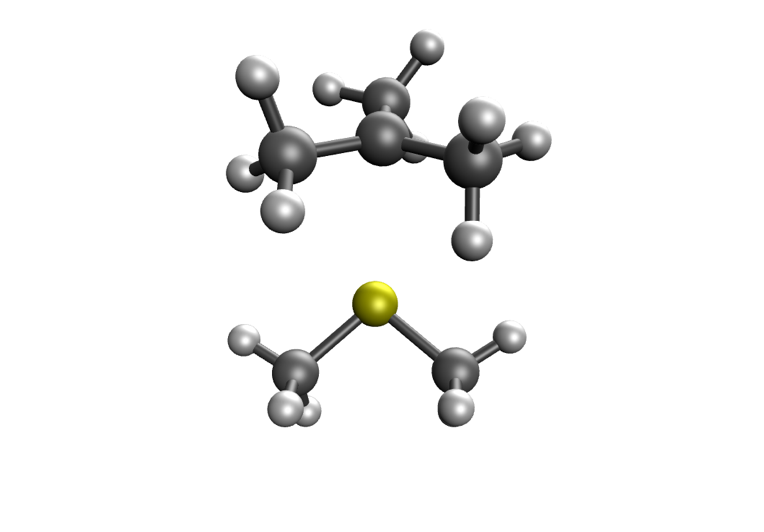

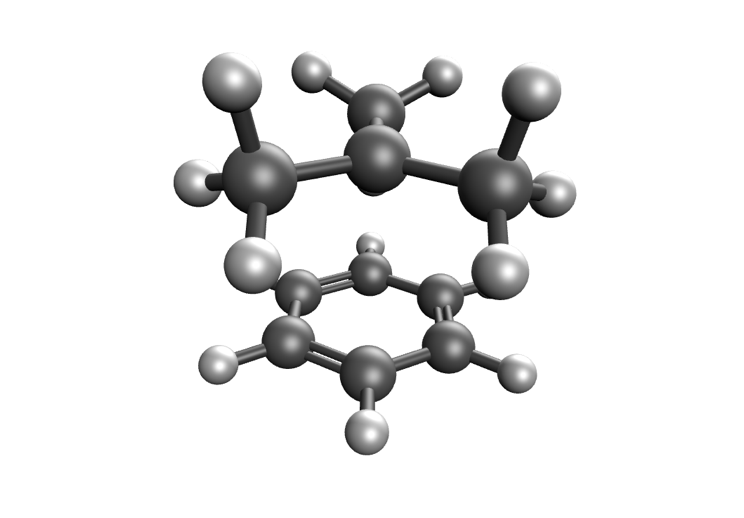

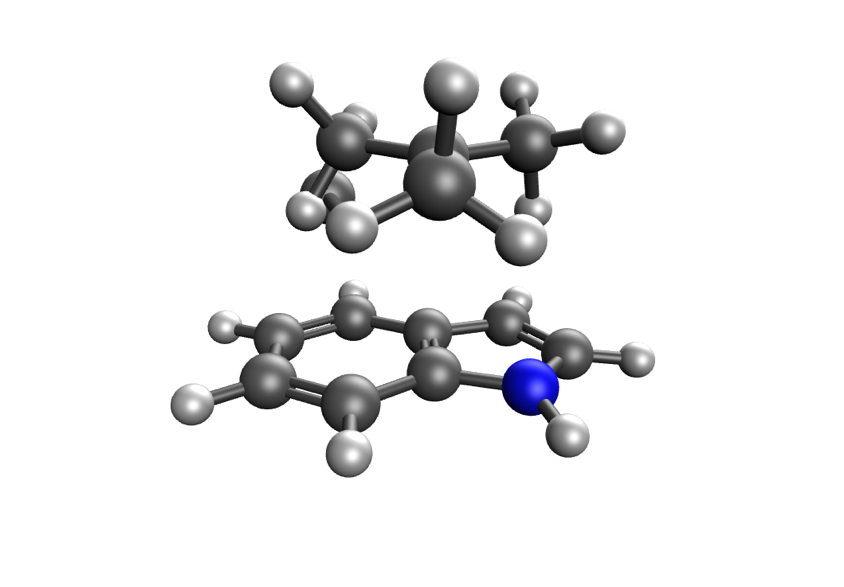
**

**Supplementary Fig. S15.** Illustrative examples of parallel interactions between (CH_3_)_3_C^+^ and dimethyl sulfide (top), benzene (middle), and indole (bottom).


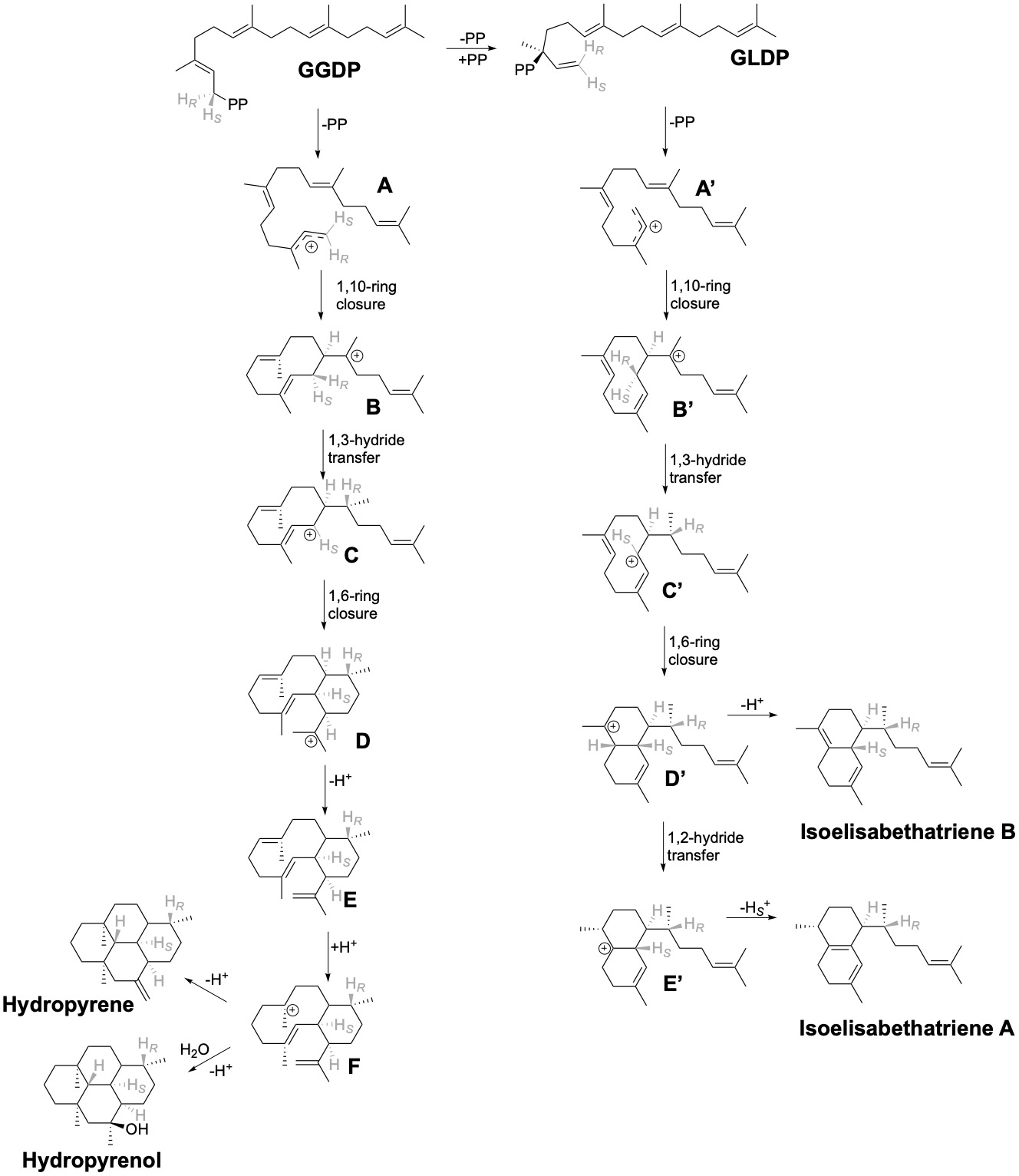


**Supplementary Scheme S1.** Suggested mechanism for hydropyrene, hyropyrenol, isoelisabethatriene A/B from reference [3].

**Supplementary Table S1.** Data collection, refinement, and validation statistics.

| **Data collection** | **Se-Met HpS^M75L^•Mg^2+^_3_•AHD** | **HpS^M75L^•Mg^2+^_3_•AHD** |
| --- | --- | --- |
| PDB entry | 8B4L | 8B4M |
| Space group | *P*2_1_2_1_2_1_ | *P*2_1_ |
| Wavelength [Å] | 0.9797 | 0.9184 |
| Unit cell a; b; c [Å] | 80.7.3; 176.7; 185.7 | 106.7; 82.3; 148.3 |
| α; β; γ [º] | 90.0; 90.0; 90.0 | 90.0; 92.3; 90.0 |
| Resolution [Å] ^a^ | 50.00 - 3.40  (3.60 - 3.40) | 50.00 - 3.04  (3.22 - 3.04) |
| Unique reflections | 70,442 (10,995) | 48,303 (7,398) |
| Completeness ^a^ | 99.3 (96.2) | 96.9 (92.8) |
| <I/σ(I)> ^a^ | 9.4 (1.1) | 5.1 (0.9) |
| R_meas_ ^a, b^ | 0.321 (2.849) | 0.310 (1.524) |
| CC_1/2_ ^a, c^ | 99.9 (62.0) | 99.5 (43.9) |
| Redundancy ^a^ | 15.6 (14.9) | 4.7 (4.7) |
| **Refinement** |  |  |
| Resolution [Å] ^a^ | 50.00 - 3.40  (3.52 - 3.40) | 50.00 - 3.04  (3.15 - 3.04) |
| Non-hydrogen atoms | 14,704 | 14,633 |
| R_work_ ^a, d^ | 0.240 (0.408) | 0.252 (0.345) |
| R_free_ ^a, e^ | 0.279 (0.454) | 0.308 (0.408) |
| No. of protein chains | 6 | 6 |
| Average B-factor [Å^2^] | 107.1 | 58.5 |
| Protein residues | 1,865 / 106.91 | 1,848 / 58.4 |
| Ligand and ions | 22 / 136.0 | 16 / 78.5 |
| r.m.s.d. ^f^ bond length [Å] | 0.003 | 0.003 |
| bond angles [°] | 0.590 | 0.69 |
| **Validation** |  |  |
| Ramachandran outliers [%] | 0.22 | 0.28 |
| Ramachandran favored [%] | 96.32 | 96.30 |
| Molprobity Clashscore | 9.98 | 7.85 |
| Molprobity Score | 1.77 | 1.69 |
| **Ramachandran plot *Z*-score, (r.m.s.d.)** |  |  |
| whole | -1.27 (0.18) | -0.67 (0.17) |
| helix | -0.22 (0.13) | 0.41 (0.12) |
| loop | -2.48 (0.26) | -2.85 (0.24) |

^a^ values in parentheses refer to the highest resolution shell.

^b^ R_meas_ = Σ_h_ [n/(n-1)]^1/2^ Σ_i_ ⏐ I_h_ - I_h,i_⏐/ Σ_h_Σ_i_ I_h,i_.where *I_h_* is the mean intensity of symmetry-equivalent reflections and *n* is the redundancy.[4]

^c^ CC_1/2_ = (<I^2^> - <I>^2^) / (<I^2^> - <I>^2^) + σ2_ε_, in which σ2_ε_ is the mean error within a half-dataset.[5]

^d^ R_work_ = Σ_h_ ⏐F_o_ – F_c_⏐/ Σ F_o_ (working set, no σ cut-off applied).

^e^ R_free_ is the same as R_work_, but calculated on 5% of the data excluded from refinement.

^f^ Root-mean-square deviation (r.m.s.d.) from target geometry.

^g^ Clashscore is the number of serious steric overlaps (> 0.4 ) per 1,000 atoms.

**Supplementary Table S2.** Modell completeness given for each protein chain. If the electron density allowed for modelling of the Mg^2+^_3_•AHD cluster, it is indicated for each protein chain.

|  | **Se-Met**  **HpS^M75L^•Mg^2+^_3_•AHD** | **Mg^2+^_3_•AHD** | **HpS^M75L^•Mg^2+^_3_•AHD** | **Mg^2+^_3_•AHD** |
| --- | --- | --- | --- | --- |
| **chain A** | 0 – 89  94 – 308 | Mg^2+^_3_•AHD | 1 – 308 | not modelled |
| **chain B** | 3 – 89  93 – 313 | Mg^2+^_3_•AHD | 2 – 37  42 – 89  95 – 317 | Mg^2+^_3_•AHD |
| **chain C** | -1 – 89  93 – 312 | Mg^2+^_3_•AHD | 0 – 39  43 – 89  93 – 307 | not modelled |
| **chain D** | 1 – 89  94 – 309 | Mg^2+^_3_•AHD | 2 – 39  43 – 87  95 – 316 | Mg^2+^_3_•AHD |
| **chain E** | 1 – 89  94 – 308 | Mg^2+^_3_•AHD | 0 – 89  93 – 310 | Mg^2+^_3_•AHD |
| **chain F** | 0 – 89  94 – 311 | Mg^2+^_1_•AHD | 1 – 89  94 – 315 | Mg^2+^_3_•AHD |

**Supplementary Table S3.** Results of a DALI search [6] with the structure of HpS^M75L^•Mg^2+^_3_•AHD.

| **PDB ID** | **rmsd [Å]** | **sequence identity [%]** | **Z-score** | **protein** | **TPS family** | **Ligand in active site** | **resolution [Å]** | **Lit.** |
| --- | --- | --- | --- | --- | --- | --- | --- | --- |
| 5dz2 | 2.2 | 24 | 32.5 | germacradienol/ geosmin synthase | sesquiterpene | alendronate, 3 Mg^2+^ | 2.11 | [7] |
| 3kb9 | 2.3 | 22 | 32.0 | epi-isozizaene synthase | sesquiterpene | PP_i_, 3 Mg^2+^, **N-benzyl-N,N-diethylethanaminium** | 1.60 | [8] |
| 4okm | 2.2 | 21 | 31.7 | selinadiene synthase | sesquiterpene | PP_i_, 3 Mg^2+^ | 2.10 | [9] |
| 5a0i | 2.4 | 24 | 31.0 | labdane-related diterpene synthase (LrdC) | diterpene | PP_i_, 2 Mg^2+^ | 2.57 |  |
| 2oa6 | 2.5 | 19 | 29.2 | aristolochene synthase | sesquiterpene | PP_i_, 3 Mg^2+^ | 2.15 | [10] |
| 4la6 | 3.6 | 17 | 28.3 | 2-methylisoborneol synthase | monoterpene | 2-fluorolinalyl diphosphate, 2 Mg^2+^ | 2.00 | [11] |
| 5i1u | 2.8 | 23 | 28.3 | germacradien-4-ol synthase | sesquiterpene | no ligand | 1.50 | [12] |
| 4xlx | 3.1 | 17 | 22.2 | *ent*-kaurene synthase (BjKS) | diterpene | no ligand | 2.00 | [13] |
| 5erm | 2.8 | 13 | 21.6 | fusicoccadiene synthase | diterpene | pamidronate, 3 Mg^2+^ | 2.30 | [14] |
| 3sae | 3.3 | 12 | 20.0 | α-bisabolene synthase | sesquiterpene | FSDP, 3 Mg^2^ | 1.96 | [15] |
| 1jfa | 4.1 | 13 | 15.7 | trichodiene synthase | sesquiterpene | no ligand | 2.50 | [16] |
| 6ggi | 3.6 | 10 | 14.2 | cyclooctat-9-en-7-ol synthase | diterpene | GGFDP, 1 Mg^2+^ | 1.80 | [1] |
| 3pyb | 3.5 | 8 | 13.1 | ent-copalyl diphosphate synthase | diterpene | 13-aza-13,14-dihydrocopalyl diphosphate | 2.76 |  |
| 5yo8 | 4.0 | 9 | 12.6 | Tetraprenyl-beta-curcumene synthase | heptaprenyl | no ligand | 1.64 | [17] |
| 5ero | 3.7 | 8 | 12.6 | fusicoccadiene synthase | diterpene | pamidronate, 3 Co^2+^ | 2.55 | [14] |
| 2dh4 | 3.6 | 8 | 12.0 | geranylgeranyl pyrophosphate synthase | diterpene | 1 Mg^2+^ | 1.98 | [18] |
| 2ewg | 3.8 | 12 | 11.9 | farnesyl pyrophosphate synthase | sesquiterpene | 1-Hydroxy-2-imidazo[1,2-a]pyridin-3-ylethane-1,1-dilyl)bis(phosphonic acid, 3 Mg^2+^ | 2.48 | [19] |

**Supplementary Table S4.** Relative proportions of IE A, IE B and HP of HpS double variant in respect to HpS^M75L^ in relation to the respective relative total terpene yields. The relative proportions of IE A, IE B and HP are displayed as percentage ratio of the relative total terpene yields. The terpene yields are calculated according to the areas of the respective GC-FID product peaks.

| HpS | | relative total  terpene yield [%] | IE A [%] | IE B [%] | HP [%] |
| --- | --- | --- | --- | --- | --- |
| M75L |  | 100.0 | 50.2 | 25.7 | 24.1 |
| M75L | M71Y | 72.0 | 41.9 | 18.3 | 11.7 |
| M75L | G182A | 93.6 | 53.8 | 21.9 | 17.9 |
| M75L | G182F | 72.1 | 24.7 | 26.8 | 20.6 |
| M75L | H184A | 32.6 | 11.2 | 11.9 | 9.5 |
| M75L | H184F | 5.6 | 0.4 | 5.0 | 0.2 |
| M75L | M300I | 74.7 | 23.9 | 45.4 | 5.3 |
| M75L | M304C | 73.0 | 7.4 | 38.4 | 27.2 |

**Supplementary Table S5.** Relative proportions of IE A, IE B and HP of HpS triple variants in respect to HpS^M75L^ and its triple variants in relation to the respective relative total terpene yields. The relative proportions of IE A, IE B and HP are displayed as percentage ratio of the relative total terpene yields. The terpene yields are calculated according to the areas of the respective GC-FID product peaks.

| HpS | | | relative total  terpene yield [%] | IE A [%] | IE B [%] | HP [%] |
| --- | --- | --- | --- | --- | --- | --- |
| M75L |  |  | 100.0 | 50.2 | 25.7 | 24.1 |
| M75L | M71Y | G182A | 116.9 | 63.5 | 27.2 | 26.2 |
| M75L | M71Y | M300I | 86.4 | 39.3 | 39.4 | 7.8 |
| M75L | M300I | M304C | 22.5 | 1.2 | 17.6 | 3.7 |

**Supplementary Table S6.** Values used to filter Interpro name column to download data matching Pfam ID: PF19086.

| **Name** |
| --- |
| Terpene_synthase  Terpene_cyclase  Terpene_synthase_family,_metal_binding_domain  Terpenoid_synthase  Terpene_synth_C_domain-containing_protein  Terpene_synth_domain-containing_protein  Terpene_synthase_family_protein  Terpenoid_synthase_protein  Terpene_synthase_18  Terpene_synthase_10  Terpene_synthase_12  Terpene_synthase_11 |

**Supplementary Table S7.** Nuclear Overhauser effect (NOE) restraints applied during EnzyDock docking. Syntax follows CHARMM.

|  | **NOE** |
| --- | --- |
| All intermediates  O-C1 distance | k_min_ 0.0 r_min_ 2.00 k_max_ 15 r_max_ 6.50 f_max_ 30 |
|  | **Hydropyrene** |
| F (C**3**-C16 distance) | k_min_ 15 r_min_ 4.1 k_max_ 0.0 r_max_ 6.50 f_max_ 30 |
| F (C**3**-C16 distance) | k_min_ 0.0 r_min_ 2.00 k_max_ 15 r_max_ 3.75 f_max_ 30 |
| G (C**3**-C17 distance) | k_min_ 15 r_min_ 4.1 k_max_ 0.0 r_max_ 6.50 f_max_ 30 |
| G (C**3**-C16 distance) | k_min_ 0.0 r_min_ 2.00 k_max_ 15 r_max_ 3.75 f_max_ 30 |

**Supplementary Table S8.** Oligonucleotides used for cloning (5’-phosporlyated primers [Pho]).

| **number** | **description** | **oligonucleotide sequence (5’ 🡪 3’)** |
| --- | --- | --- |
| 1 | M75L_M71Y fwd | [Pho]TATACCATTCTGTTGGCCTGGTATGCAGAATATG |
| 2 | M75L_M71Y rev | [Pho]AAATGCCAGGGTATCCCAGCTTGCATTTTC |
| 3 | M75L_M300I fwd | [Pho]ATTATGTGGTCAATGGTTGATTGGAGCGCAC |
| 4 | M75L_M300I rev | [Pho]ATCTTCCAGAAAACGAACATGACGACCTGTTG |
| 5 | M75L_M304C fwd | [Pho]TGCGTTGATTGGAGCGCACGTAGTG |
| 6 | M75L_M304C rev | [Pho]TGACCACATCATATCTTCCAGAAAACGAACATGAC |
| 7 | M75L_G182F fwd | [Pho]TTTGGTCATGTTTATGGTATGTATATTCTGGGTGCAG |
| 8 | M75L_G182F rev | [Pho]ATGACGACGCATACCAATATGTGCGCTC |
| 9 | M75L_G182A fwd | [Pho]GCGGGTCATGTTTATGGTATGTATATTCTGGG |
| 10 | M75L_H184F fwd | [Pho]TTTGTTTATGGTATGTATATTCTGGGTGCAGCCG |
| 11 | M75L_H184F rev | [Pho]ACCACCATGACGACGCATACCAATATGTG |
| 12 | M75L_H184A fwd | [Pho]GCGGTTTATGGTATGTATATTCTGGGTGCAG |
| 13 | M75L_M300I_M304C rev | [Pho]TGACCACATAATATCTTCCAGAAAACGAACATGAC |

**Supplementary Table S9.** QM(M06-2X)/MM energies (kcal/mol) for the reactions leading to HP and HPol in HpS^WT^ and HpS^M75L^. Gas phase values from ref. [20] are added for reference.

| **Intermediate** | **HpS^WT^(QM/MM)** | **HpS^M75L^(QM/MM)** | **Gas-phase** |
| --- | --- | --- | --- |
| **A** | 0.0 | 0.0 | 0.0 |
| **B** | -4.9 | -18.6 | -11.4 |
| **C** | -30.4 | -35.2 | -18.5 |
| **I** | -82.9 | -87.0 | -62.0 |

**Supplementary Table S10.** QM(M06-2X)/MM energies (kcal/mol) for the reactions leading to IE A and IE B in HpS^WT^ and HpS^M75L^. Gas phase values from ref. [20] are added for reference.

| **Intermediate** | **HpS^WT^(QM/MM)** | **HpS^M75L^(QM/MM)** | **Gas-phase** |
| --- | --- | --- | --- |
| **A'** | 0.0 | 0.0 | 0.0 |
| **B'** | -2.0 | -1.1 | -15.7 |
| **C'** | -25.1 | -30.3 | -32.1 |
| **D'** | -39.9 | -31.8 | -35.9 |
| **E'** | -55.6 | -59.4 | -34.4 |

**Supplementary Table S11.** Model calculations of the complexation energy (Δ*E^C^*) between dimethyl sulfide (DMS) with cation **B** (HP pathway) and cation **B’** (IE pathway).*^a^* The complexation energy was calculated as Δ*E^C^ = E^B-DMS^ –* (*E^B^ + E^DMS^*) where B is the cation B or B’, and *E^B^* and *E^DMS^* are the electronic energies of individual cation and DMS molecules.

|  | **complexation energy [kcal/mol]** | **distance [Å]** |
| --- | --- | --- |
| **B gas phase** | -29.11 | 1.91 |
| **B chloroform** | -28.39 |  |
| **B water** | -30.14 |  |
| **B' gas phase** | -25.29 | 1.92 |
| **B' chloroform** | -24.95 |  |
| **B' water** | -26.24 |  |

*^a^* Method: M06-2X/6-31+G(d,p).

**Supplementary Table S12.** Model calculations of the complexation energy (kcal/mol) between fragments benzene, indole, and dimethyl sulfide with model cations in gas-phase, chloroform, and water (parallel orientation).

|  | **Interaction Energy (kcal/mol)** *^a^* | | | **Interaction Distance (Å)** *^b^* | | | |
| --- | --- | --- | --- | --- | --- | --- | --- |
|  | **Gas-phase** | **Chloroform** | **Water** | **Gas-phase** | **Chloroform** | **Water** |  |
| **Benzene-CH_3_^+^** | -86.99 | -64.83 | -59.37 | 1.547 | 1.540 | 1.538 |  |
| **Benzene-CH_3_CH_2_^+^** | -46.39 | -30.46 | -26.54 | 1.558 | 1.546 | 1.543 |  |
| **Benzene-(CH_3_)_2_CH^+^** | -28.65 | -16.98 | -14.16 | 1.586 | 1.562 | 1.557 |  |
| **Benzene-(CH_3_)_3_C^+^** | -13.77 | -6.15 | -4.62 | 3.151 | 3.200 | 3.212 |  |
| **Indole-CH_3_^+^** | -109.40 | -82.35 | -75.57 | 1.571 | 1.543 | 1.542 |  |
| **Indole-CH_3_CH_2_^+^** | -67.43 | -46.81 | -41.62 | 1.591 | 1.563 | 1.561 |  |
| **Indole-(CH_3_)_2_CH^+^** | -49.91 | -34.11 | -30.10 | 1.581 | 1.573 | 1.571 |  |
| **Indole-(CH_3_)_3_C^+^** | -20.57 | -9.18 | -6.87 | 3.124 | 3.192 | 3.181 |  |
| **(CH_3_)_2_S-CH_3_^+^** | -115.23 | -96.31 | -91.61 | 1.790 | 1.786 | 1.785 |  |
| **(CH_3_)_2_S-CH_3_CH_2_^+^** | -75.33 | -61.74 | -58.33 | 1.812 | 1.807 | 1.805 |  |
| **(CH_3_)_2_S-(CH_3_)_2_CH^+^** | -58.91 | -49.00 | -46.50 | 1.833 | 1.827 | 1.826 |  |
| **(CH_3_)_2_S-(CH_3_)_3_C^+^** | -44.49 | -36.53 | -34.51 | 1.857 | 1.852 | 1.850 |  |
| **(CH_3_)_2_S-(CH_3_)_3_C^+^ *^c^*** | -12.40 | -7.47 | -5.97 | 3.115 | 3.115 | 3.115 |  |

*^a^* The complexation electronic energy was calculated as Δ*E = E^C-F^ –* (*E^C^ + E^F^*) where C is the model cation and F the fragment. Method: $\omega$B97M-V/def2-TZVPD.  *^b^* Distance was computed as the nearest C-C distance for benzene and indole containing complexes and C-S distance for dimethyl sulfide complexes. *^c^* Complex computed at a non-stationary point with a distance of 3.115 Å.

**Supplementary Table S13.** Model SAPT2 non-covalent interaction energy calculations (kcal/mol).

|  | **Benzene-(CH_3_)_3_C^+^ *^a^*** | **Indole-(CH_3_)_3_C^+^ *^b^*** | **(CH_3_)_2_S-CH_3_^+^ *^c^*** |
| --- | --- | --- | --- |
| E1_elst | -11.351 | -17.360 | -13.474 |
| E1_exch | 15.437 | 23.334 | 13.806 |
| E2_ind (resp) | -11.060 | -14.317 | -14.426 |
| E2_exch-ind (resp) | 5.857 | 7.311 | 9.463 |
| E2_disp | -12.358 | -17.850 | -8.495 |
| E2_exch-disp | 1.487 | 2.121 | 1.345 |
| Total of energy components | -11.988 | -16.761 | -11.782 |

*^a^* Closest C^+^-C atom-atom distance: 3.124 Å (local minimum). *^b^* Closest C^+^-C atom-atom distance (local minimum): 3.151 Å. *^c^* C^+^-S atom-atom distance: 3.115 Å (non-stationary point).

**Supplementary Movie S1.** HpS^M75L^ adopts the classical α‐fold of class I TPSs, but it is composed of nine instead of the common ten core α‐helices. Instead, the ten residues at the N-terminus of HpS^M75L^ adopt a coiled structure that meanders on the protein surface. Catalytic residues lining the active site are shown as sticks in cyan. The aspartate-rich motif ^82^**DD**RAI**D**^87^ is highlighted in red and the ^225^NSE^233^ motif in yellow. Mg^2+^ ions are shown as green spheres. The AHD molecule is presented as a ball and stick model with carbon atoms colored in black, oxygen in red, phosphorous in orange and nitrogen in blue.

**References**

1. Driller, R., Janke, S., Fuchs, M., Warner, E., Mhashal, A. R., Major, D. T., Christmann, M., Bruck, T. & Loll, B. (2018) Towards a comprehensive understanding of the structural dynamics of a bacterial diterpene synthase during catalysis, *Nat Commun.* **9**, 3971.

2. Liebschner, D., Afonine, P. V., Moriarty, N. W., Poon, B. K., Sobolev, O. V., Terwilliger, T. C. & Adams, P. D. (2017) Polder maps: improving OMIT maps by excluding bulk solvent, *Acta Crystallogr D Biol Crystallogr.* **73**, 148-157.

3. Rinkel, J., Rabe, P., Chen, X., Kollner, T. G., Chen, F. & Dickschat, J. S. (2017) Mechanisms of the Diterpene Cyclases β-Pinacene Synthase from *Dictyostelium discoideum* and Hydropyrene Synthase from *Streptomyces clavuligerus*, *Chemistry.* **23**, 10501-10505.

4. Diederichs, K. & Karplus, P. A. (1997) Improved R-factors for diffraction data analysis in macromolecular crystallography, *Nat Struct Biol.* **4**, 269-75.

5. Karplus, P. A. & Diederichs, K. (2012) Linking crystallographic model and data quality, *Science.* **336**, 1030-3.

6. Holm, L. & Rosenstrom, P. (2010) Dali server: conservation mapping in 3D, *Nucleic Acids Res.* **38**, W545-9.

7. Harris, G. G., Lombardi, P. M., Pemberton, T. A., Matsui, T., Weiss, T. M., Cole, K. E., Koksal, M., Murphy, F. V. t., Vedula, L. S., Chou, W. K., Cane, D. E. & Christianson, D. W. (2015) Structural Studies of Geosmin Synthase, a Bifunctional Sesquiterpene Synthase with aa Domain Architecture That Catalyzes a Unique Cyclization-Fragmentation Reaction Sequence, *Biochemistry.* **54**, 7142-55.

8. Aaron, J. A., Lin, X., Cane, D. E. & Christianson, D. W. (2010) Structure of epi-isozizaene synthase from *Streptomyces coelicolor* A3(2), a platform for new terpenoid cyclization templates, *Biochemistry.* **49**, 1787-97.

9. Baer, P., Rabe, P., Fischer, K., Citron, C. A., Klapschinski, T. A., Groll, M. & Dickschat, J. S. (2014) Induced-fit mechanism in class I terpene cyclases, *Angew Chem Int Ed Engl.* **53**, 7652-6.

10. Shishova, E. Y., Di Costanzo, L., Cane, D. E. & Christianson, D. W. (2007) X-ray crystal structure of aristolochene synthase from *Aspergillus terreus* and evolution of templates for the cyclization of farnesyl diphosphate, *Biochemistry.* **46**, 1941-51.

11. Köksal, M., Chou, W. K., Cane, D. E. & Christianson, D. W. (2013) Unexpected reactivity of 2-fluorolinalyl diphosphate in the active site of crystalline 2-methylisoborneol synthase, *Biochemistry.* **52**, 5247-55.

12. Grundy, D. J., Chen, M., Gonzalez, V., Leoni, S., Miller, D. J., Christianson, D. W. & Allemann, R. K. (2016) Mechanism of Germacradien-4-ol Synthase-Controlled Water Capture, *Biochemistry.* **55**, 2112-21.

13. Liu, W., Feng, X., Zheng, Y., Huang, C. H., Nakano, C., Hoshino, T., Bogue, S., Ko, T. P., Chen, C. C., Cui, Y., Li, J., Wang, I., Hsu, S. T., Oldfield, E. & Guo, R. T. (2014) Structure, function and inhibition of ent-kaurene synthase from *Bradyrhizobium japonicum*, *Sci Rep.* **4**, 6214.

14. Chen, M., Chou, W. K., Toyomasu, T., Cane, D. E. & Christianson, D. W. (2016) Structure and Function of Fusicoccadiene Synthase, a Hexameric Bifunctional Diterpene Synthase, *ACS Chem Biol.* **11**, 889-99.

15. McAndrew, R. P., Peralta-Yahya, P. P., DeGiovanni, A., Pereira, J. H., Hadi, M. Z., Keasling, J. D. & Adams, P. D. (2011) Structure of a three-domain sesquiterpene synthase: a prospective target for advanced biofuels production, *Structure.* **19**, 1876-84.

16. Rynkiewicz, M. J., Cane, D. E. & Christianson, D. W. (2001) Structure of trichodiene synthase from Fusarium sporotrichioides provides mechanistic inferences on the terpene cyclization cascade, *Proc Natl Acad Sci U S A.* **98**, 13543-8.

17. Fujihashi, M., Sato, T., Tanaka, Y., Yamamoto, D., Nishi, T., Ueda, D., Murakami, M., Yasuno, Y., Sekihara, A., Fuku, K., Shinada, T. & Miki, K. (2018) Crystal structure and functional analysis of large-terpene synthases belonging to a newly found subclass, *Chem Sci.* **9**, 3754-3758.

18. Chang, T. H., Guo, R. T., Ko, T. P., Wang, A. H. & Liang, P. H. (2006) Crystal structure of type-III geranylgeranyl pyrophosphate synthase from Saccharomyces cerevisiae and the mechanism of product chain length determination, *J Biol Chem.* **281**, 14991-5000.

19. Mao, J., Mukherjee, S., Zhang, Y., Cao, R., Sanders, J. M., Song, Y., Zhang, Y., Meints, G. A., Gao, Y. G., Mukkamala, D., Hudock, M. P. & Oldfield, E. (2006) Solid-state NMR, crystallographic, and computational investigation of bisphosphonates and farnesyl diphosphate synthase-bisphosphonate complexes, *J Am Chem Soc.* **128**, 14485-97.

20. Zev, S., Ringel, M., Driller, R., Loll, B., Bruck, T. & Major, D. T. (2022) Understanding the competing pathways leading to hydropyrene and isoelisabethatriene, *Beilstein J Org Chem.* **18**, 972-978.
